# Differential proinflammatory activities of Spike proteins of SARS-CoV-2 variants of concern

**DOI:** 10.1126/sciadv.abo0732

**Published:** 2022-09-16

**Authors:** Sylwia D. Tyrkalska, Alicia Martínez-López, Ana B. Arroyo, Francisco J. Martínez-Morcillo, Sergio Candel, Diana García-Moreno, Pablo Mesa-del-Castillo, María L. Cayuela, Victoriano Mulero

**Affiliations:** ^1^ Departmento de Biología Celular e Histología, Facultad de Biología, Universidad de Murcia, 30100 Murcia, Spain.; ^2^ Instituto Murciano de Investigación Biosanitaria (IMIB)-Arrixaca, 30120 Murcia, Spain.; ^3^ Centro de Investigación Biomédica en Red de Enfermedades Raras (CIBERER), Instituto de Salud Carlos III, 28029 Madrid, Spain.; ^4^ Hospital Clínico Universitario Virgen de la Arrixaca, 30120 Murcia, Spain.

## Abstract

The coronavirus disease 2019 (COVID-19) pandemic turned the whole world upside down in a short time. One of the main challenges faced has been to understand COVID-19–associated life-threatening hyperinflammation, the so-called cytokine storm syndrome (CSS). We report here the proinflammatory role of Spike (S) proteins from different severe acute respiratory syndrome coronavirus 2 (SARS-CoV-2) variants of concern in zebrafish. We found that wild-type/Wuhan variant S1 (S1WT) promoted neutrophil and macrophage recruitment, local and systemic hyperinflammation, emergency myelopoiesis, and hemorrhages. In addition, S1γ was more proinflammatory S1δ was less proinflammatory than S1WT, and, notably, S1β promoted delayed and long-lasting inflammation. Pharmacological inhibition of the canonical inflammasome alleviated S1-induced inflammation and emergency myelopoiesis. In contrast, genetic inhibition of angiotensin-converting enzyme 2 strengthened the proinflammatory activity of S1, and angiotensin (1-7) fully rescued S1-induced hyperinflammation and hemorrhages. These results shed light into the mechanisms orchestrating the COVID-19–associated CSS and the host immune response to different SARS-CoV-2 S protein variants.

## Teaser

Zebrafish reveals the differential proinflammatory activities of Spike proteins of SARS-CoV-2 variants of concern.

## INTRODUCTION

By the end of 2019, a new viral disease–causing pneumonia had been reported in Wuhan, China (*1*). Metagenomic RNA sequencing revealed that a new betacoronavirus called severe acute respiratory syndrome coronavirus 2 (SARS-CoV-2) was responsible for the outbreaks in China and soon in the rest of the world. The disease was named coronavirus disease 2019 (COVID-19) and was announced as the second pandemic of the 21st century by the World Health Organization on 11 March 2020 (*2*).

COVID-19 is believed to be transmitted via respiratory droplets and fomites. When the viral units are inhaled into the respiratory tract through the nasopharyngeal mucosal membranes, SARS-CoV-2 binds to the epithelial cells and starts replicating and migrating down to the airways reaching the alveolar epithelial cells in the lungs (*3*). SARS-CoV-2 infection in humans varies and may manifest itself as mild symptoms to severe respiratory failure. The most common symptoms of COVID-19 include fever, dry cough, malaise, myalgia, vomiting, diarrhea, and abdominal pain (*3*, *4*). Moreover, neurological, musculoskeletal and cardiovascular failures have lately been included in a list of potential COVID-19 complications as a result of multiple organ failure that can even lead to death (*5*). Although the clinical manifestations of COVID-19 differ with age, all ages of the population are susceptible to SARS-CoV-2 infection (*6*). The first signs of the disease become evident after an incubation period of 1 to 14 days, most commonly around day 5 (*5*).

The phylogenetic analysis of the whole genome of a novel SARS-CoV-2 virus shows close similarity with SARS-related coronaviruses (*7*). It has been shown that it shares 79% genome sequence identity with SARS-CoV and 50% with Middle East respiratory syndrome coronavirus (*8*). SARS-CoV-2 consists of a long single-stranded positive-sense RNA molecule, which is surrounded by a lipid envelope that anchors many structural viral glycoproteins (*9*). The viral genome is organized in 10 open reading frames (ORFs) encoding nonstructural polyproteins (67%) and accessory or structural proteins (33%). Four major structural proteins are encoded by SARS-CoV-2 genome: spike (S), envelope (E), membrane (M), and nucleocapsid (N) (*10*). S protein has lately become the most studied structural protein among the viral proteins due to its role in viral recognition by the host and ability to mutate, thus generating new SARS-CoV-2 variants.

S protein is a trimeric glycoprotein that is encoded by ORF2 in the viral genome. It is formed by a membrane-distal S1 subunit and a membrane-proximal S2 subunit, which form homotrimers in the virus envelope (*11*). The S1 subunit recognizes the host cellular receptor angiotensin-converting enzyme 2 (ACE2) via its receptor-binding domain (RBD) located at the C terminus, while its N terminus is composed of N-terminal domain (*11*). The S2 subunit is essential for the membrane fusion that initiates virus entry. Recognition of the host cell receptor is followed by the structural rearrangements of SARS-CoV-2 S protein. More specifically, RBD domain, which is situated in the external subdomain, opens to bind to the ACE2 protein in the host cell (*11*). This results in S protein binding to two more ACE2 proteins to form the S protein bound to three ACE2 proteins. Subsequently, the S protein complex is cleaved by furin and transmembrane serine protease 2 (TMPRSS2) to release the ACE2-S1 fragments (*11*). Proteolytical cleavage can happen at two cleavage sites to separate S1 and S2 (*11*). As the S2 domain remains and is already primed for viral entry into the host cell, the refolding process of the spring-loaded S2 subunit initiates host and viral membranes fusion.

Viral genomes can undergo adaptive mutations that may provoke alterations in the virus’ pathogenic potential. It has been seen that even a single amino acid exchange can markedly affect and perturb the ability of a virus to evade the host immune system and hence complicate the progression of vaccine development against the virus (*12*). Unfortunately, SARS-CoV-2, similar to other RNA viruses, is prone to genetic evolution and develops mutations over time, resulting in the emergence of multiple variants that may have different characteristics compared to their ancestral strains (*12*, *13*). Of the many different variants of SARS-CoV-2 that have evolved to date, this study concentrates on the three of most immediate concern and particular interest: Beta (lineage B.1.351), Gamma (lineage P.1), and Delta (lineage B.1.617.2) (*13*).

SARS-CoV-2 Beta variant was first detected in South Africa in October 2020, showing many new mutations especially in the region of S protein. It was observed that this variant spread more rapidly, and its prevalence was higher among young people with no underlying health conditions and frequently resulted in serious illness. Moreover, the Beta variant was able to attach to human cells more easily because of three mutations in the RBD within S protein: N501Y, K417N, and E484K (*14*). The SARS-CoV-2 Gamma variant arose in Brazil in early 2021, showing 17 unique amino acid changes, 10 of which were in its S protein, including the three mutations of most concern, N501Y, E484K, and K417T, which change the stability of RBD-hACE2 complex affecting the binding affinity of RBD to human ACE2 (hACE2) (*15*, *16*). Gamma variant infections are 2.2 times more transmissible, have the same ability to infect both adults and older persons, and can produce a 10-fold increase in viral load compared to persons infected by other variants (*14*). The SARS-CoV-2 Delta variant was first found in India in late 2020, mutations in the S protein causing substitutions in T478K, L452R, and P681R. These mutations are known to affect the transmissibility of the virus, and it is thought to be one of the most transmissible respiratory viruses known to date; in addition, they are also involved in immunoescape (*14*, *17*, *18*).

To date, the zebrafish model is one of the fastest growing animal models used for reproducing all types of human diseases, such as cardiovascular diseases, infectious diseases, cancer, neurodegeneration diseases, hematopoietic disorders, and many more. Its small size, large number of eggs generated at a time, and external fertilization, the transparency of the embryos, rapid development, and easy maintenance in the laboratory make the zebrafish an attractive model for research in many fields (*19*). Moreover, genetics and physiologic similarities with mammals increase their value as a powerful tool (*19*). Since the beginning of COVID-19 pandemic, many laboratories have started modeling the disease in zebrafish. Although zebrafish does not have lungs, they have a hindbrain ventricle, a cavity filled with cerebrospinal fluid into which immune cells can be recruited. This is a convenient injection site to study host and viral factors involved in local and whole-body immune response and innate immunity in general (*20*). Here, we describe a zebrafish model to study the immune response driven by different S variants of SARS-CoV-2 and explain the different proinflammatory activities that contribute to COVID-19–associated cytokine storm syndrome (CSS) (*21*).

## RESULTS

### Wild-type S1 is highly proinflammatory in zebrafish

**Fig. 1.**
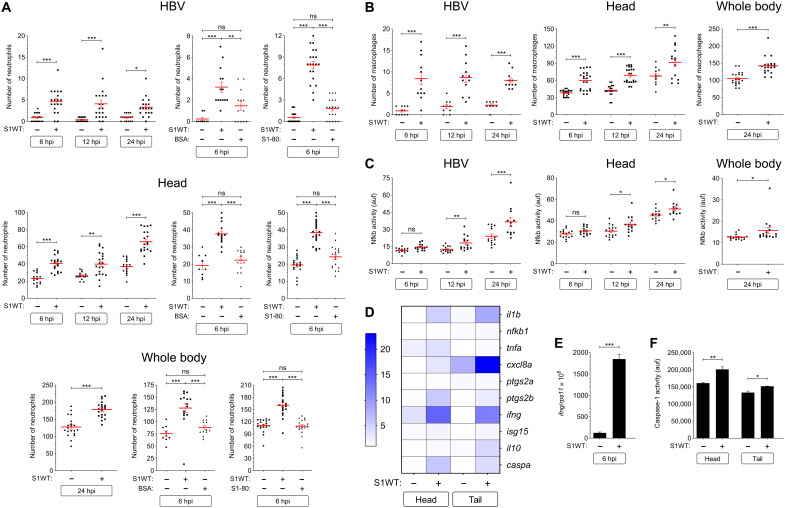
WT S1 is highly proinflammatory in zebrafish. Recombinant S1WT (+), BSA, or vehicle (−) were injected in the hindbrain ventricle (HBV) of 2–day postfertilization (dpf) *Tg(mpx:eGFP)* (**A**), *Tg(mfap4:mCherry)* (**B**), *Tg(*nfkb*:eGFP)* (**C**), and WT (**D** to **F**) larvae. Neutrophil (A) and macrophage (B) recruitment and number and Nfkb activation (C) were analyzed at 6, 12, and 24 hpi by fluorescence microscopy, the transcript levels of the indicated genes (D) were analyzed at 12 hpi by RT-qPCR in larval head and tail (except for *ifng* that was also analyzed at 6 hpi in the head) (E), and caspase-1 activity was determined at 24 hpi using a fluorogenic substrate (F). As a control, S1WT was also used preheated at 80°C for 30 min (S1-80). Each dot represents one individual, and the means ± SEM for each group is also shown. *P* values were calculated using one-way analysis of variance (ANOVA) and Tukey multiple range test. RT-qPCR data are depicted as a heatmap in (D) with higher expression shown in darker color. ns, not significant; *P ≤ 0.05, **P ≤ 0.01, and ***P ≤ 0.001. auf, arbitrary units of fluorescence.

Here, we present a novel zebrafish model for COVID-19 using the recombinant S1 domain from wild-type (WT) S protein (S1WT) injected into the hindbrain of 48–hour postfertilization (hpf) zebrafish larvae. The model does not need the virus to be used for the infection, which makes it easier, safer, and cheaper to study the COVID-19–associated CSS. Injection of S1WT induced a robust recruitment of innate immune cells, namely, neutrophils (*mpx:eGFP* or *lyz:dsRED2*) and macrophages (*mfap4:mcherry*), to the site of the injection. By 6 hours postinjection (hpi), both neutrophils and macrophages were present in the hindbrain of S1WT-injected larvae, in contrast to the control larvae injected with water, bovine serum albumin (BSA), or preheated S1WT, where the numbers of both cell types were insignificant (Fig. 1, A and B, and fig. S1, A and B). This difference remained until 12 and 24 hpi, suggesting that S1WT can be recognized by the zebrafish immune system (Fig. 1, A and B). The same pattern can be seen by counting the number of neutrophils and macrophages in the head of the injected larvae (Fig. 1, A and B, and fig. S1, A and B). Moreover, the total number of neutrophils and macrophages in the whole body was higher after S1WT injection compared with their control siblings at 24 hpi. This indicates that S1WT injected into the hindbrain was able to activate emergency hematopoiesis leading to increased production of neutrophils and macrophages.

To check how the inflammation process proceeds in the fish injected with S1WT, we first analyzed the expression pattern of the master regulator of the inflammatory response nuclear factor κB (Nfkb) using the reporter line *nfkb:eGFP*. Although, at 6 hpi, there was no increase in the fluorescent signal levels of Nfkb in the site of the injection or the head, increased fluorescence started to appear at 12 and continued until 24 hpi (Fig. 1C and fig. S1C). Nfkb had not only increased locally but a slight increase was also seen globally in the whole body at 24 hpi (Fig. 1C and fig. S1C). These results suggest that S1WT is able to not only initiate the immune response locally at the site of the injection in zebrafish but also cause a systemic inflammation that spreads throughout the zebrafish body.

To confirm these results, we used reverse transcription quantitative polymerase chain reaction (RT-qPCR) to measure the transcript levels of proinflammatory genes in the head (locally) and rest of the body/tail (systemically) at 12 hpi. The transcript levels of *il1b* (interleukin-1beta), *tnfa* (tumor necrosis factor alpha), *cxcl8a* (chemokine C-X-C motif ligand 8a), *ptgs2a* (rostaglandin-endoperoxide synthase 2a), and *ptgs2b* increased locally in the head, whereas *il1b*, *nfkb1* (nuclear factor of kappa light polypeptide gene enhancer in B-cells 1), *cxcl8a*, *ptgs2b*, and *isg15* (ISG15 ubiquitin like modifier) also increased in the rest of the body, further confirming the systemic inflammation triggered by S1WT (Fig. 1D and fig. S2, A to H). Curiously, the mRNA levels of the gene encoding interferon-γ (Ifng related) were seen to have markedly increased at 6 hpi, but they returned to basal levels at 12 hpi (Fig. 1E and fig. S2G). The mRNA levels of the gene encoding the antiinflammatory cytokine Il10 (interleukin-10) increased systemically in the S1WT injected larvae (Fig. 1D and fig. S2I).

Recently, it has been shown that inflammasome plays an important role in the SARS-CoV-2 infection (*22*). To check whether the injection of S1WT into the hindbrain of the zebrafish larvae is able to activate the expression of genes other than inflammatory genes, RT-qPCR was used to measure the transcript levels of caspase a (Caspa; the functional homolog of human CASP1) The *caspa* transcript levels increased at both local and systemic levels (Fig. 1D and fig. S2J). Moreover, caspase-1 activity also increased in both the head and the rest of the body of S1WT-injected larvae (Fig. 1F).

### Full-length S protein phenocopies the effects of S1 protein in zebrafish

To verify the results obtained with the zebrafish model for COVID-19 using recombinant S1WT protein injected into the hindbrain, we expressed WT full-length S by forcing expression of its RNA, which was injected into the yolk of one-cell-stage embryos. Using this strategy, full-length S was ubiquitously expressed as a membrane-anchored protein, as occurs in infected cells. Using this model, we observed Nfkb induction (fig. S3A) and higher transcript levels of *il1b*, *nfkb1*, *tnfa*, *cxcl8a*, *ptgs2a* and *ptgs2b*, *infg*, and *il10* in the larvae expressing full-length S (fig. S3, B to I) compared with control larvae. However, *isg15* mRNA levels were unaltered in response to full-length S (fig. S3J). Although the mRNA levels of the genes encoding the most important inflammasome components Pycard (apoptosis-associated speck-like protein containing a CARD, also known as Asc) and Caspa were not affected by the forced expression of full-length S (fig. S3, K and L), caspase-1 activity was higher in these fish than in the controls (fig. S3M). Collectively, these results confirmed the strong proinflammatory effects of WT S in zebrafish larvae.

### Recombinant S1, S1 + S2, and E proteins are all proinflammatory in zebrafish

Initially, it was believed that only S protein, especially its S1 domain, was responsible for host immune system recognition, forming part of the outer sheath of the virus (*23*). However, E protein, which is only believed to play a role in the viral assembly, also forms part of this sheath facing outward, and is recognized by Toll-like receptor 2 (TLR2) to promote inflammation (*24*). We therefore injected the hindbrain of zebrafish larvae with recombinant S1, S1 + S2, or E proteins from the WT variant and checked neutrophil and macrophage recruitment to the site of the injection 6, 12, and 24 hpi. The results showed that S1, S1 + S2, and E were all able to recruit both type of immune cells to the site of the injection at similar levels (fig. S4, A and B). Moreover, the number of neutrophils and macrophages in the head of the fish injected with S1, S1 + S2, and E were similar, whereas the total number of neutrophils and macrophages in the whole body increased only in the larvae injected with either S1 or S1 + S2 (fig. S4, A and B). This suggests that S1 and S1 + S2, but not E protein, were able to induce emergency myelopoiesis, as further confirmed by the ability of S1 and S1 + S2 to induce *csf3a* transcript levels (fig. S4C). Very probably, the S1 domain was responsible for the induction of emergency myelopoiesis since the presence of S2 did not further increase it.

All viral proteins used in this experiment were able to increase Nfkb activity in hindbrain, head, and whole body, all to a similar extent over the levels of their control siblings at all time points tested (fig. S4D). This was further confirmed by RT-qPCR experiments, whereby S1, S1 + S2, and E were all able to induce similar transcript levels of *il1b*, *nfkb1*, *tnfa*, *cxcl8a*, *il10*, *infg*, *ptgs2a*, ptgs2b, *caspa*, and *pycard* (fig. S4, E to N). However, E protein systemically induced *il1b*, *cxcl8a*, *ptgs2a*, and *ptgs2b* transcript levels, whereas S1 + S2 induced more robustly those of *tnfa*, *infg*, and *il10* (fig. S4, E to N). Caspase-1 activity was increased by all viral proteins locally and systemically but E protein in particular (fig. S4O).

### S1WT signals through the canonical inflammasome in zebrafish

**Fig. 2.**
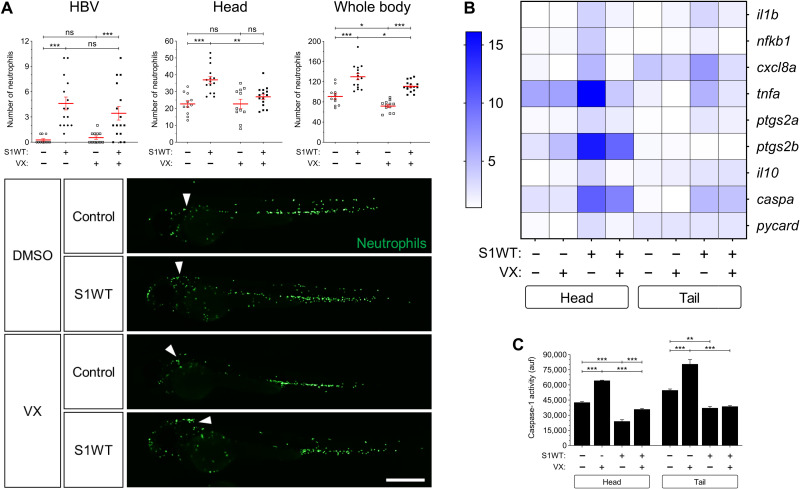
WT S1 signals through the canonical inflammasome in zebrafish. Recombinant S1WT or vehicle (−) were injected in the hindbrain ventricle (arrowheads) of 2-dpf *Tg(mpx:eGFP)* (**A**) and WT (**B** and **C**) larvae in the presence of either dimethyl sulfoxide (DMSO) or the caspase-1 inhibitor VX-765 (VX). Neutrophil recruitment and number were analyzed at 3 hpi by fluorescence microscopy (A), the transcript levels of the indicated genes were analyzed at 12 hpi by RT-qPCR (B), and caspase-1 activity was determined at 24 hpi using a fluorogenic substrate (C). Representative images of whole *Tg(mpx:eGFP)* larvae for each treatment are also shown (A). Each dot represents one individual and the means ± SEM for each group is also shown. RT-qPCR data are depicted as a heatmap in (B) with higher expression shown in darker color. *P* values were calculated using one-way ANOVA and Tukey multiple range test. *P ≤ 0.05, **P ≤ 0.01, and ***P ≤ 0.001. Scale bar, 500 μm.

The inflammasome also plays a pivotal role in COVID-19 (*22*). To ascertain whether this pathway also mediates the proinflammatory activity of S1WT in zebrafish, the larvae were treated with the specific caspase-1 inhibitor, VX-765, by bath immersion. Although the pharmacological inhibition of caspase-1 failed to inhibit the recruitment of neutrophils to the hindbrain (Fig. 2A), it was able to decrease the number of neutrophils in the head and in the whole body (Fig. 2A). This result suggests that S1WT rapidly activated emergency myelopoiesis via the canonical inflammasome in zebrafish.

Next, we checked the transcript levels of proinflammatory genes in heads and the rest of the body of the fish injected with S1WT and treated with VX-765. It was found that pharmacological inhibition of the canonical inflammasome robustly decreased the induction of the proinflammatory genes caused by the injection of S1WT into the hindbrain of the zebrafish larvae (Fig. 2B and fig. S5). In addition, *il1b*, *nfkb1*, and *cxcl8a* mRNA levels decreased at both local and systemic levels upon VX-765 treatment (Fig. 2B and fig. S5, A to C), whereas those of *tnfa*, *ptgs2a*, *ptgs2b*, and *il10* decreased only at the injection site (Fig. 2B and fig. S5, D to G). Although no differences in *caspa* transcript levels were seen when S1WT-injected larvae were treated with VX-765 (Fig. 2B and fig. S5H), those of *pycard* decreased to basal levels upon VX-765 treatment (Fig. 2B and fig. S5I). As expected, VX-765 decreased caspase-1 activity in the control and S1WT-injected larvae (Fig. 2C). All these results indicate that SARS-CoV-2 S protein activates the canonical inflammasome in zebrafish.

### Ace2 deficiency exacerbates the proinflammatory activity of S1WT in zebrafish

**Fig. 3.**
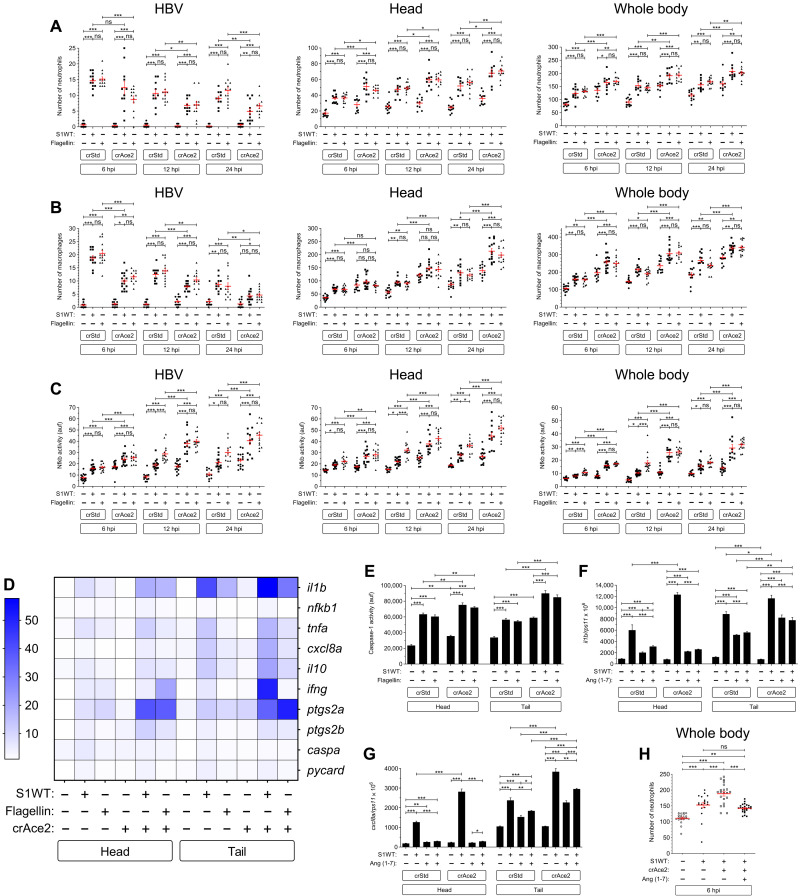
Ace2 deficiency exacerbates the proinflammatory activity of S1WT in zebrafish. One-cell-stage zebrafish eggs of *Tg(mpx:eGFP)* (**A** and **H**), *Tg(mfap4:mCherry)* (**B**), *Tg(*nfkb*:eGFP)* (**C**), and WT (**D** to **G**) were microinjected with control or *ace2* crRNA-Cas9 complexes. At 2 dpf, recombinant S1WT, flagellin, or vehicle (−) was injected alone or in combination with Ang (1-7) in the hindbrain ventricle of control and Ace2-deficient larvae. Neutrophil (A and H) and macrophage (B) recruitment and number and Nfkb activation (C) were analyzed at 6, 12, and 24 hpi by fluorescence microscopy; the transcript levels of the indicated genes were analyzed at 12 hpi by RT-qPCR (D, F, and G); and caspase-1 activity was determined at 24 hpi using a fluorogenic substrate (E). Each dot represents one individual and the means ± SEM for each group is also shown. RT-qPCR data are depicted as a heatmap in (D) with higher expression shown in darker color. *P* values were calculated using one-way ANOVA and Tukey multiple range test. *P ≤ 0.05, **P ≤ 0.01, and ***P ≤ 0.001.

ACE2 is a zinc-containing metalloenzyme attached to the cell membrane, which is involved in the regulation of blood pressure through the hydrolysis of angiotensin-II (Ang-II) into angiotensin (1-7) [Ang (1-7)] (*25*). Moreover, ACE2 serves as the entry point into the cells for different coronaviruses, among others SARS-CoV-2 (*26*). Thus, we decided to check whether Ace2 was playing any role in the activation of zebrafish innate immunity mediated by recombinant S1WT protein. RT-qPCR analysis showed that *ace2* was expressed in both head and tail, although higher transcript levels were observed in the latter and were induced by S1WT (fig. S6A). We found that although Ace2 deficiency (fig. S6B) does not affect larval development, as in mice (*27*), it resulted in reduced neutrophil and macrophage recruitment to the injection site in both S1WT- and flagellin-injected larvae. In contrast, Ace2-deficient larvae showed higher number of neutrophils and macrophages in the head and in whole body than WT larvae at all time points analyzed upon S1WT injection (Fig. 3, A and B). Similarly, Nfkb activity was also higher in Ace2-deficient larvae injected with either S1WT or flagellin at the injection site, but also systemically, than in their control siblings (Fig. 3C). It is worth mentioning that even the basal level of Nfkb in Ace2-deficient larvae injected with water was elevated in hindbrain, head, and the whole body (Fig. 3C).

The key anti-inflammatory role of Ace2 in zebrafish was further confirmed by the local and systemic increased transcript levels of *il1b*, nfkb1, *tnfa*, *cxcl8a*, *il10*, ifng, *ptgs2a*, and *ptgs2b* upon S1WT injection and, to some extent, flagellin injection (Fig. 3D and fig. S7, A to H). Curiously, Ace2 deficiency failed to alter *caspa* and *pycard* mRNA levels (Fig. 3D and fig. S7, I and J) and caspase-1 activity (Fig. 3E) upon S1WT or flagellin injection. Notably, the injection of Ang (1-7) together with S1WT fully rescued the local hyperinflammation observed in Ace2-deficient larvae, as determined by quantitating the transcript levels of *il1b* and *cxcl8a* (Fig. 3, F and G) and neutrophilia (Fig. 3H) at 6 hpi in whole larvae.

### S1γ variant is more proinflammatory than the S1WT

**Fig. 4.**
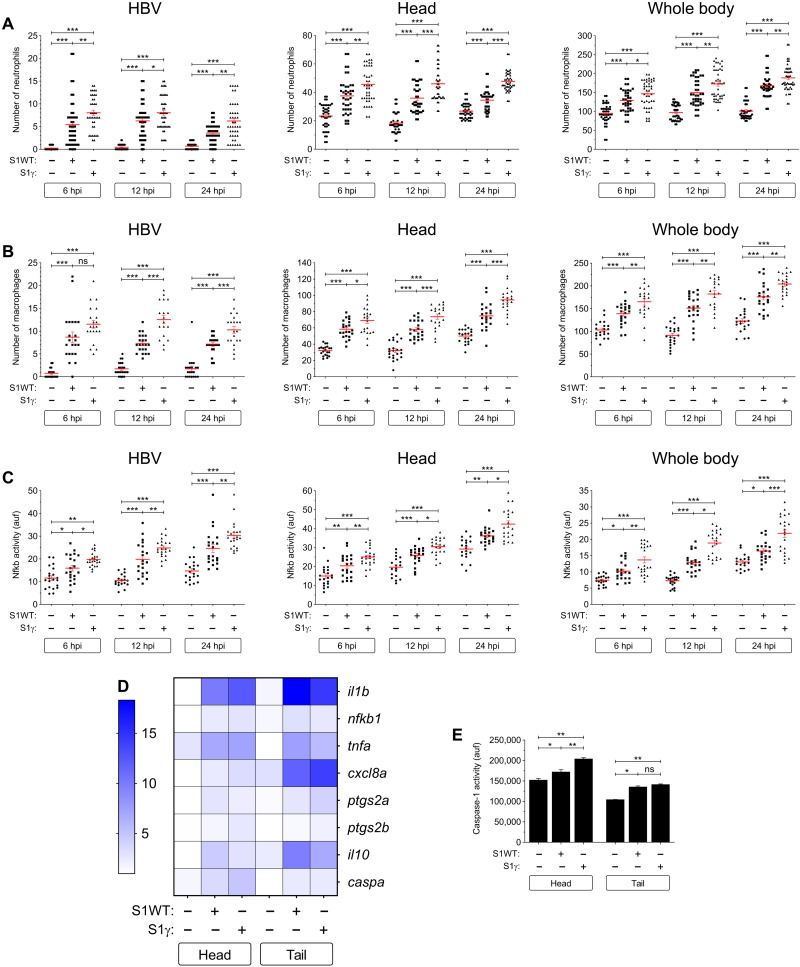
S1γ variant is more proinflammatory than the S1WT. Recombinant S1WT, S1γ, or vehicle (−) were injected in the hindbrain ventricle of 2-dpf larvae of *Tg(mpx:eGFP)* (**A**), *Tg(mfap4:mCherry)* (**B**), *Tg(*nfkb*:eGFP)* (**C**), and WT (**D** and **E**). Neutrophil (A) and macrophage (B) recruitment and number and Nfkb activation (C) were analyzed at 6, 12, and 24 hpi by fluorescence microscopy; the transcript levels of the indicated genes were analyzed at 12 hpi by RT-qPCR (D); and caspase-1 activity was determined at 24 hpi using a fluorogenic substrate (E). Each dot represents one individual and the means ± SEM for each group is also shown. RT-qPCR data are depicted as a heatmap in (D) with higher expression shown in darker color. *P* values were calculated using one-way ANOVA and Tukey multiple range test. *P ≤ 0.05, **P ≤ 0.01, and ***P ≤ 0.001.

We next checked in vivo whether there were any differences in the proinflammatory activities of recombinant S1WT and S1γ. It was found that fish injected with S1γ showed higher recruitment and a greater number of neutrophils and macrophages at all time points compared with the S1WT (Fig. 4, A and B). Similarly, the S1γ also induced higher Nfkb activity more rapidly (Fig. 4C) and, to some extent, proinflammatory gene transcripts (Fig. 4D and fig. S8, A to F) than S1WT. Notably, S1γ induced lower mRNA levels of *il10* both locally and systemically compared with S1WT (Fig. 4D and fig. S8G). Moreover, S1γ increased higher transcript levels of *caspa* (Fig. 4D and fig. S8H) and caspase-1 activity (Fig. 4E). All these results suggest that S1γ is more proinflammatory and activates emergency myelopoiesis more robustly than S1WT.

### S1δ is less proinflammatory than the S1WT

**Fig. 5.**
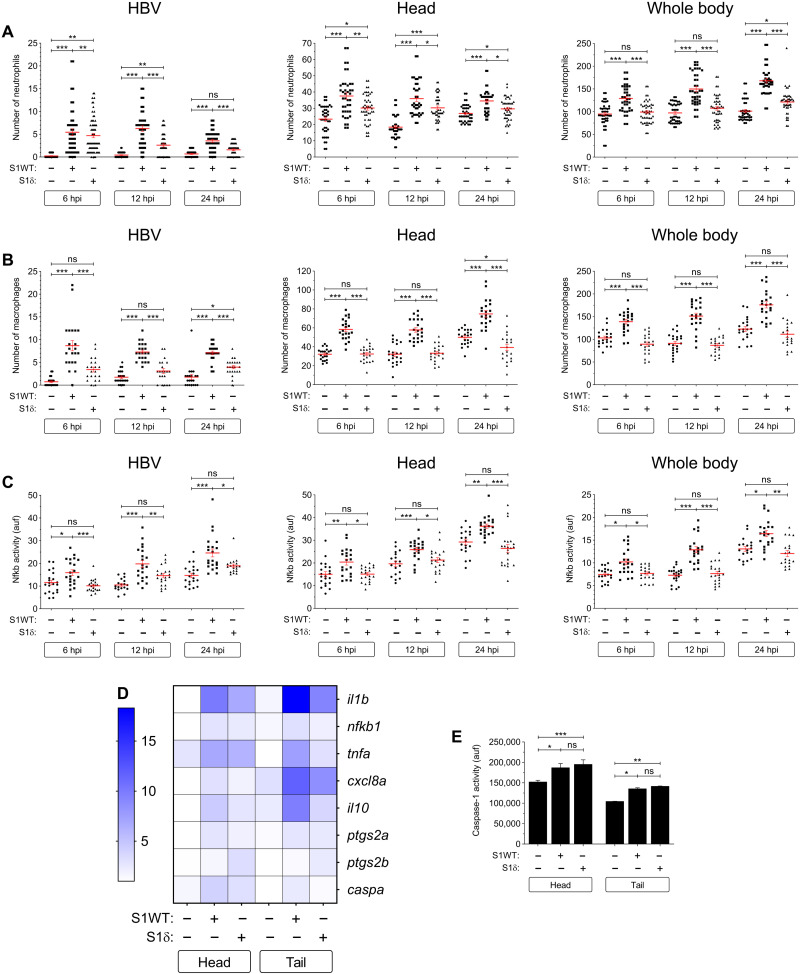
S1δ is less proinflammatory than the S1WT. Recombinant S1WT, S1δ, or vehicle (−) were injected in the hindbrain ventricle of 2-dpf larvae of *Tg(mpx:eGFP)* (**A**), *Tg(mfap4:mCherry)* (**B**), *Tg(*nfkb*:eGFP)* (**C**), and WT (**D** and **E**). Neutrophil (A) and macrophage (B) recruitment and number and Nfkb activation (C) were analyzed at 6, 12, and 24 hpi by fluorescence microscopy; the transcript levels of the indicated genes were analyzed at 12 hpi by RT-qPCR (D); and caspase-1 activity was determined at 24 hpi using a fluorogenic substrate (E). Each dot represents one individual and the means ± SEM for each group is also shown. RT-qPCR data are depicted as a heatmap in (D) with higher expression shown in darker color. *P* values were calculated using one-way ANOVA and Tukey multiple range test. *P ≤ 0.05, **P ≤ 0.01, and ***P ≤ 0.001.

As regard S1δ, we found that the recruitment of neutrophils and macrophages to the injection site was less robust in larvae injected with S1δ than with S1WT, while their total numbers were comparable with the control larvae injected with water (Fig. 5, A and B). Similarly, S1δ failed to induce Nfkb at any of the time points tested (Fig. 5C) and was less potent at inducing the mRNA levels of inflammatory genes, except for *ptgs2b* (Fig. 5D and fig. S9, A to H). However, S1W and S1δ induced caspase-1 activity at similar levels (Fig. 5E). Collectively, these results suggest that S1δ is much less proinflammatory than S1WT and fails to activate emergency hematopoiesis.

### S1β shows delayed but stronger proinflammatory activity than the S1WT

**Fig. 6.**
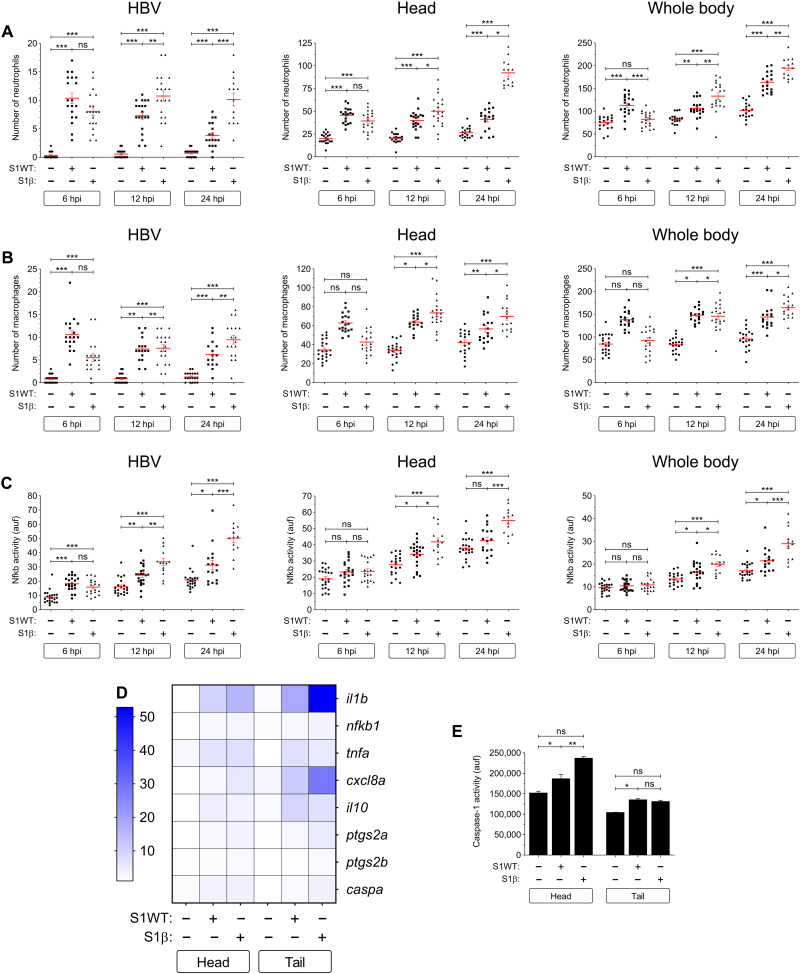
S1β shows delayed but stronger proinflammatory activity than the S1WT. Recombinant S1WT, S1β, or vehicle (−) were injected in the hindbrain ventricle of 2-dpf larvae of *Tg(mpx:eGFP)* (**A**), *Tg(mfap4:mCherry)* (**B**), *Tg(*nfkb*:eGFP)* (**C**), and WT (**D** and **E**). Neutrophil (A) and macrophage (B) recruitment and number and Nfkb activation (C) were analyzed at 6, 12, and 24 hpi by fluorescence microscopy; the transcript levels of the indicated genes were analyzed at 12 hpi by RT-qPCR (D); and caspase-1 activity was determined at 24 hpi using a fluorogenic substrate (E). Each dot represents one individual and the means ± SEM for each group is also shown. RT-qPCR data are depicted as a heatmap in (D) with higher expression shown in darker color. *P* values were calculated using one-way ANOVA and Tukey multiple range test. *P ≤ 0.05, **P ≤ 0.01, and ***P ≤ 0.001.

It was observed that, although the initial influx (6 hpi) of neutrophils and macrophages to the injection site in response to S1β and S1WT was similar, or even slower for S1β, myeloid cell recruitment markedly increased at 12 and 24 hpi in larvae injected with S1β (Fig. 6, A and B). The same delayed induction pattern was observed in S1β-injected larvae for the Nfkb activity (Fig. 6C), while the transcript levels of inflammatory genes were higher in S1β-injected larvae than their S1WT-injected counterparts, except for *tnfa* and *il10*, at 12 hpi (Fig. 6D and fig. S10, A to G). In addition, S1β induced higher *caspa* mRNA levels (Fig. 6D and fig. S10H) and caspase-1 activity in the injection site than S1WT (Fig. 6E).

To further confirm that S1β was able to induce a sustained inflammatory response, *il1b* and *cxcl8a* mRNA levels were analyzed at 24 and 48 hpi. The results confirmed that, at 24 hpi, the larvae injected with S1β had higher *il1b* and *cxcl8a* levels than those injected with S1WT (fig. S11A). However, at 48 hpi, the transcript levels of these genes had returned to basal levels in both S1WT and S1β (fig. S11B). These results show that S1β has delayed but longer-lasting proinflammatory effects compared with the rest of S1 variants of concern (VOCs).

### Recombinant S1 proteins provoke hemorrhages in zebrafish larvae

**Fig. 7.**
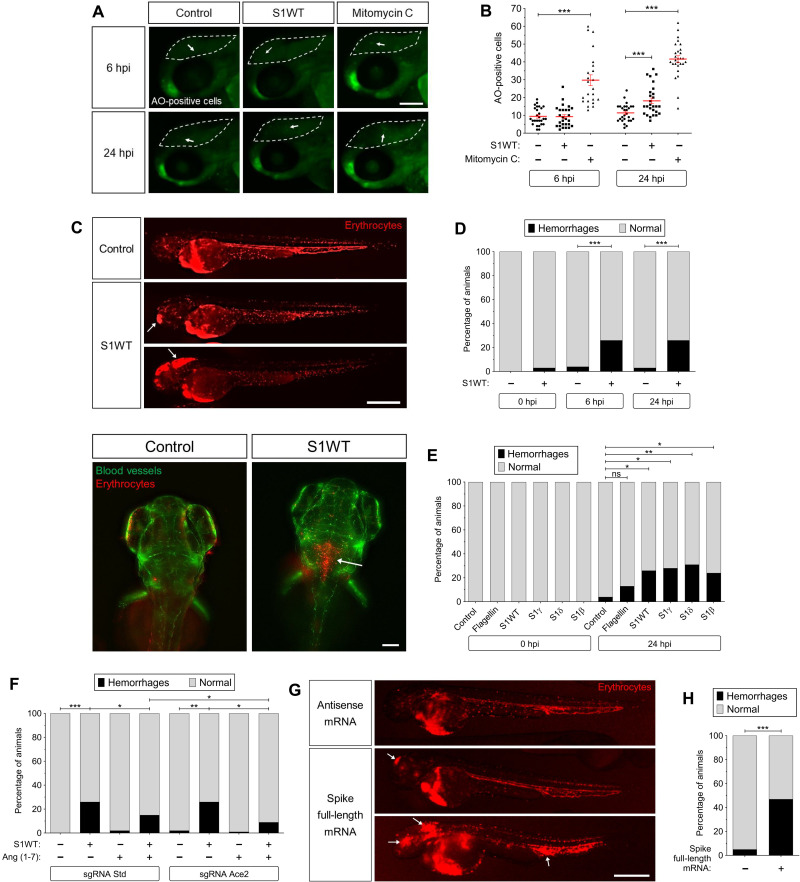
Recombinant S1 proteins provoke hemorrhages in zebrafish larvae. (**A** and **B**) WT larvae were injected with S1WT protein, mitomycin C, or vehicle (−) in the hindbrain ventricle at 2 dpf and the number of cell death in the brain analyzed at 6 and 24 hpi by acridine orange (AO) staining. (**C** to **F**) One-cell-stage zebrafish eggs of *Tg(gata1a:dsRed)*/*Tg(fli1a:eGFP)* were microinjected with control or *ace2* crRNA-Cas9 complexes. At 2 dpf, S1 proteins or flagellin were injected in the hindbrain ventricle alone or with Ang (1-7). Hemorrhages were analyzed at 0, 6, and 24 hpi. (**G** and **H**) Zebrafish eggs of *Tg(gata1a:dsRED)* were microinjected with control or full-length S RNAs, and hemorrhages were analyzed at 48 hpf. Representative images showing AO^+^ cells (A) and hemorrhages (white arrows) (C and G). *P* values were calculated using one-way ANOVA and Tukey multiple range test (B) and Fisher’s exact test (D, E, G, and H). (D) 0 hpi + H_2_O, *n* = 34; 0 hpi + S1WT, *n* = 50; 6 hpi + H_2_O, *n* = 50; 6 hpi + S1WT, *n* = 60; 24 hpi + H_2_O, *n* = 52; and 24 hpi + S1WT, *n* = 60. (E) 0 hpi + H_2_O, 35; 0 hpi + flagellin, *n* = 35; S1WT, *n* = 47; S1γ, *n* = 40; S1δ, *n* = 42; S1β, *n* = 46. (F) crSTD + H_2_O, *n* = 92; crSTD + S1WT, *n* = 85; crSTD + ANG (1-7), *n* = 83; crSTD + S1WT + ANG (1-7), *n* = 87; crAce2 + H_2_O, *n* = 76; crAce2 + S1WT, *n* = 83; crAce2 + ANG (1-7), *n* = 90; crAce2 + S1WT + ANG (1-7), *n* = 81. *P ≤ 0.05, **P ≤ 0.01, and ***P ≤ 0.001. Scale bars, 100 μm (A), 500 μm (top of C, and G), 50 μm (bottom of C).

It has been noticed that patients with COVID-19 may present an increased risk of bleeding and develop different types of hemorrhages, mostly intracerebrally, with devastating consequences (*28*–*30*). We, therefore, analyzed whether the injection of recombinant S1 proteins may produce hemorrhages using a zebrafish line with labeled erythrocytes, i.e., *tg(gata1a:dsRed)*. Notably, although the injection of S1WT protein into the hindbrain ventricle failed to promote cell death in the injection site at 6 hpi (Fig. 7, A and B), it was able to induce hemorrhages in the head of 25% of larvae at this time point, remaining until 24 hpi (Fig. 7, C and D). All the S1 variants used gave rise to comparable percentages of larvae with hemorrhages (~25%) (Fig. 7E), while flagellin injection hardly induced hemorrhage (Fig. 7E). As Ace2 and its catalytic product, Ang (1-7), were able to alleviate the proinflammatory effect of S1 in zebrafish larvae, we tested their impact on S1-induced hemorrhages. The results showed that although the genetic inhibition of Ace2 did not increase the percentage of larvae with hemorrhages triggered by S1WT, Ang (1-7) strongly decreased the percentage of larvae showing hemorrhages (Fig. 7F). The ability of recombinant S1 proteins to give rise to hemorrhages in zebrafish rather than the injection itself was further confirmed by injecting full-length RNA of WT S into one-cell-stage embryos, leading to hemorrhages in the head of about 45% of the larvae at 48 hpf (Fig. 7, G and H).

### Human mononuclear cells and neutrophils failed to respond to recombinant S proteins

**Fig. 8.**
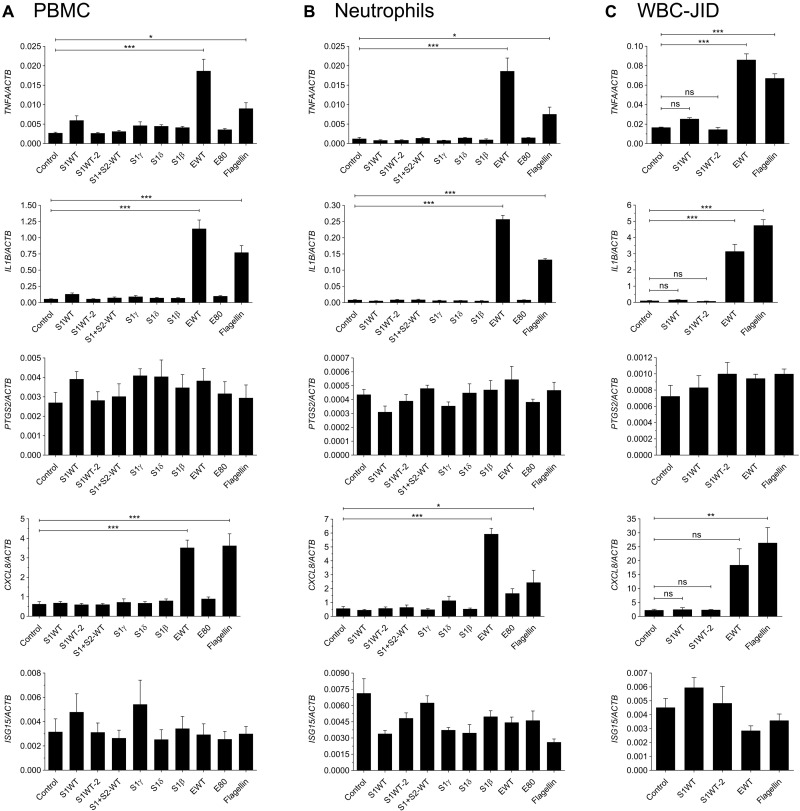
Human mononuclear cells and neutrophils failed to respond to recombinant S proteins. PBMCs (**A**) and neutrophils (**B**) from healthy donors and white blood cells (WBC) from the synovial fluid of a patient with recent onset oligoarticular juvenile idiopathic arthritis (JID) (**C**) were stimulated for 4 hours with recombinant S1 (1 μg/ml; WT, γ, δ, and β), S1 + S2 (WT), and E (WT) proteins, and the mRNA levels of the indicated genes analyzed by RT-qPCR. S1WT from Sino Biological (S1WT) and ABclonal (S1WT-2) were used. As a control, recombinant E protein was used preheated at 80°C for 30 min (E80). Data are shown as the means ± SEM of three technical replicates from two (A and B) or one (C) donors. *P* values were calculated using one-way ANOVA, followed by Tukey multiple range test. *P ≤ 0.05, **P ≤ 0.01, and ***P ≤ 0.001.

The results obtained in zebrafish prompted us to analyze the impact of recombinant S1 proteins in human white blood cells, since controversial data have been reported in this respect. Unexpectedly, all recombinant S1 proteins tested failed to induce the transcript levels of genes encoding *TNFA*, *IL1B*, *PTGS2*, *CXCL8*, and *ISG15* in peripheral blood mononuclear cells (PBMCs) (Fig. 8A) and neutrophils (Fig. 8B). We also used S1WT from another manufacturer, as well as S1 + S2, and they also failed to stimulate PBMCs (Fig. 8A) and peripheral blood neutrophils (Fig. 8B). In contrast, recombinant E protein induced the same mRNA levels of all tested genes, except *ISG15*, with a similar potency as flagellin in both cell types (Fig. 8, A and B). As it has been reported that human macrophages need to be primed to respond to recombinant S (*31*), we next used white blood cells obtained from the synovial fluid of a patient with recent onset oligoarticular juvenile idiopathic arthritis. The results showed that although these cells were primed and flagellin and recombinant E protein induced higher transcript levels of *TNFA*, *IL1B*, *PTGS2*, and *CXCL8* than in PBMCs and neutrophils, they were unable to respond to recombinant S proteins (Fig. 8C).

## DISCUSSION

The SARS-CoV-2 S protein is highly immunogenic, and current COVID-19 vaccines are based on this property. However, there is controversy about the innate immune receptors and the signaling mechanisms involved in its recognition. While several studies have reported that S protein is able to interact and activate TLR4 in human THP-1 and HL-60 cells (*32*) and murine macrophages and microglia (*32*–*34*), others failed to demonstrate the activation of human or murine macrophages by S protein (*24*). In addition, an elegant study has recently shown that S protein signals through TLR2 and activates the NLRP3 (NLR family pyrin domain containing 3) inflammasome in human macrophages obtained from convalescent patients with COVID-19, but not from healthy SARS-CoV-2–naïve individuals (*31*). Although this study reveals that SARS-CoV-2 infection causes profound and long-lived reprogramming of macrophages resulting in augmented immunogenicity of the SARS-CoV-2 S protein, it did not answer the question concerning the inability of naïve macrophages to respond to S proteins, despite expressing TLR2, which, however, was able to respond to zymosan (*31*) and, intriguingly, to E protein (*24*). We have also found that PBMCs and circulating neutrophils from healthy individuals failed to respond to S proteins, despite being able to recognize and mount an innate immune response to flagellin and E protein. Similarly, primed white blood cells, obtained from the synovial fluid of a patient with recent onset oligoarticular juvenile idiopathic arthritis, were also unable to respond to S protein, making these observations even more puzzling. In contrast, we found that zebrafish larvae were able to mount a quick innate immune response to recombinant S1 and S1 + S2 obtained from different commercial sources, as well as to full-length S protein ubiquitously expressed in the larvae upon mRNA injection. Therefore, this model circumvents the limitations and discrepancies obtained with human and mouse macrophages concerning the immunogenic and proinflammatory properties of SARS-CoV-2 S protein and shed light into the COVID-19–associated CSS. Thus, we observed that S protein induced not only the local and systemic expression of genes encoding proinflammatory mediators but also NFκB activation, neutrophilia, and monocytosis. To the best of our knowledge, this is the first study showing that S protein is able to induce a marked neutrophilia and monocytosis, which are both involved in the pathogenesis of COVID-19 (*35*) and may be of relevance for the efficacy and side effects of S-based vaccines. A wide array of hematopoietic adverse effects associated with mRNA COVID-19 vaccines was reported whose etiology need to be clarified (*36*).

Although the inflammasome has been found to participate in COVID-19, a mechanistic understanding of its involvement in COVID-19 progression remains unclear (*22*). Clinical trials with Anakinra, a modified human IL-1 receptor antagonist approved to treat rheumatoid arthritis, provided mixed results on patient benefits (*22*). We found that pharmacological inhibition of caspase-1, the effector of the canonical inflammasome, strongly alleviated the proinflammatory activity of S protein in zebrafish larvae. This effect was observed at two different levels: (i) the dampening of gene encoding proinflammatory mediators used in our study as a surrogate of the COVID-19–associated CSS and (ii) the alleviation of neutrophilia. However, no differences in neutrophil recruitment to S protein were found between untreated and VX-765–treated larvae, indicating that the inflammasome was not involved in this process, as has been previously reported using a *Salmonella enterica* serovar Typhimurium infection model, where Cxcl8 and leukotriene B4 mediate neutrophil recruitment, while the inflammasome is responsible for the bacterial clearance (*37*). The impact of the inhibition of the inflammasome in both the S protein–induced inflammation and neutrophilia is an important observation, since dysregulated hematopoiesis has been observed in patients with severe COVID-19 (*38*); moreover, the canonical inflammasome regulates the erythroid/myeloid decision of hematopoietic stem and progenitor cells (HSPCs) via cleavage of the master erythroid transcription factor GATA binding protein 1 (GATA1) (*39*). Although E protein was also highly proinflammatory in zebrafish, it failed to promote emergency myelopoiesis, suggesting that E protein is unable to activate the inflammasome in HSPCs, despite its ability to activate the NLRP3 inflammasome in mouse macrophages (*24*). Therefore, the zebrafish model developed here may contribute to understanding the contribution of the inflammasome to the CSS and the dysregulated hematopoiesis observed in severe patients with COVID-19 and to further understand the specific contribution of different viral structural proteins, as well as to identify therapeutic targets and novel drugs to treat COVID-19.

ACE2 is critical in the pathogenesis of COVID-19, not only as the viral receptor that allows the virus to invade the cells but also as a major regulator of the renin angiotensin system (RAS) (*40*). However, the role of ACE2 and RAS in COVID-19 is controversial. It has been hypothesized that viral infection promotes ACE2 internalization and down-regulation following viral entry, promoting lower levels of Ang (1-7) but higher levels of Ang-II, which is linked to inflammation and fibrosis (*40*). However, no substantial changes in circulating RAS peptides or peptidases were found between uninfected and patients with moderate COVID-19 (*41*). Ace2 inhibition exacerbated the proinflammatory effects of S protein in zebrafish larvae, and the administration of Ang (1-7) rescued the hyperinflammatory effects induced by S protein in Ace-2–deficient larvae. A similar effect of the Ace2/Ang (1-7) axis was observed in larvae injected with flagellin, suggesting a broad and strong anti-inflammatory effect of Ace2-derived Ang (1-7) rather than the neutralization of S protein by zebrafish Ace2. This is also supported by the inability of SARS-CoV-2 to replicate in zebrafish (*42*, *43*). Furthermore, although Ace2 deficiency had no impact on S1-induced hemorrhages, the administration of Ang (1-7) was able to reduce the percentage of S1-injected larvae showing hemorrhages. These results are of clinical relevance, since SARS-CoV-2 infection also induces vascular complications (*44*) and Ang (1-7) has been found to be a useful therapeutic target for the treatment of cardiovascular disease, especially in patients with overactive RAS (*45*). Our results point to a greater relevance of the ACE2/Ang (1-7)/MAS receptor axis than of the ACE/Ang-II/angiotensin type 1 receptor axis on COVID-19–associated CSS and vascular complications and to the therapeutic potential of Ang (1-7) in this disease.

**Fig. 9.**
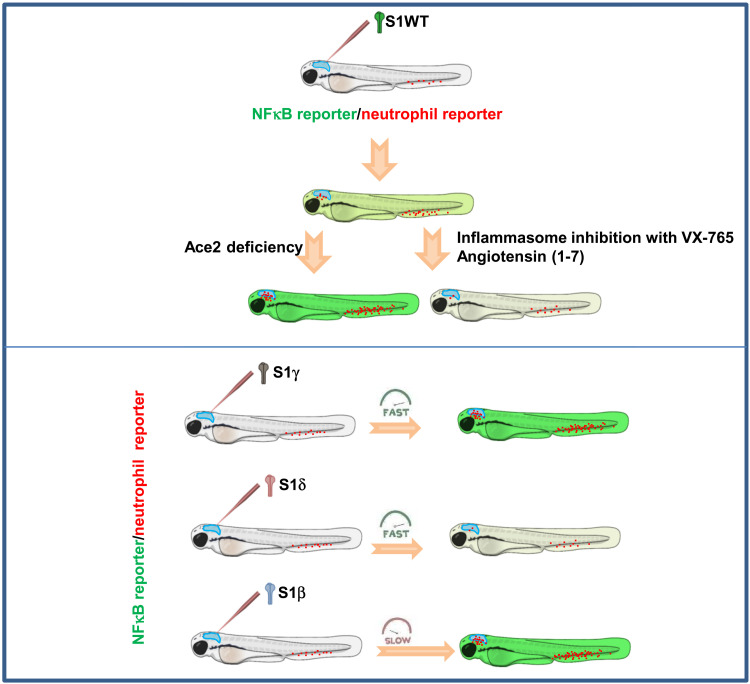
Model showing the differential activities of the S protein of SARS-CoV-2 VOCs. S1 proteins promote neutrophil and macrophage recruitment, local and systemic hyperinflammation, emergency myelopoiesis, and hemorrhages. Inhibition of Ace2 exacerbates all these effects, while inhibition of the inflammasome with the caspase-1 inhibitor VX-765 or Ang (1-7) treatment alleviates them. S1γ was more proinflammatory, while S1δ was less proinflammatory than S1WT, and S1β promoted delayed and long-lasting inflammation.

One of the most interesting observations of our study is the marked differences observed among the VOCs used (Fig. 9). S1γ was found to be much more proinflammatory than S1WT, whereas S1δ showed an opposite behavior. Notably, S1δ failed to induce emergency myelopoiesis, despite being able to induce caspase-1 activity at similar levels as S1WT. As the inhibition of caspase-1 attenuated S1WT-induced emergency myelopoiesis, other mechanisms may be involved, such as the regulation of myeloid colony-stimulating factors by S proteins. Another possibility is that S1δ activates an inflammasome not involved in the regulation of hematopoiesis. Whatever the outcome, it is tempting to speculate that the reduced proinflammatory activity of S1δ reported here would facilitate high viral loads (~1000 times higher) to be reached in the early stages of infection (*46*), together with its enhanced infectivity via cell surface entry facilitated by its increased efficiency to cleave the full-length S to S1 and S2 (*47*) and to fuse membranes at low levels of cellular receptor ACE2 (*48*). As regard the S1β, we unexpectedly observed a delayed, but long-lasting, proinflammatory activity. Although the relevance of this observation in the contagiousness and pathogenesis of the SARS-CoV-2 Beta VOC remains to be determined, it has been reported that both Alpha and Beta VOCs produce tissue-specific cytokine signatures pathogenic patterns distinct from early strains in K18-hACE2 transgenic mice models (*49*).

In summary, our zebrafish model developed to study COVID-19–associated CSS and the impact of different VOCs of SARS-CoV-2 S proteins suggests that the canonical inflammasome and the ACE2/Ang (1-7) axis are key signaling pathways involved in the recognition of S protein by the host. In addition, the lower proinflammatory activity of S1δ may explain the high viral load reached by this VOC of SARS-CoV-2 in the early stages of infection. This model, therefore, is an excellent platform for the chemical screening of anti-inflammatory compounds to alleviate COVID-19–associated CSS.

## MATERIALS AND METHODS

### Animals

Zebrafish (*Danio rerio* H.) were obtained from the Zebrafish International Resource Center and mated, staged, raised, and processed as described (*50*). The lines *Tg(mpx:eGFP)^i114^* (*51*), *Tg(lyz:DsRED2)^nz50^* (*52*), *Tg(mfap4:mCherry-F)^ump6^* (*53*), *Tg(*nfkb*:eGFP)^sh235^* referred to as *nfkb:eGFP* (*54*), *Tg(gata1a:DsRed)^sd2^* (*55*), *Tg(fli1:EGFP)*^*y*1^ (*56*), and casper (*mitfa^w2/w2^; mpv17^a9/a9^)* (*57*) were previously described. The experiments performed comply with the Guidelines of the European Union Council (Directive 2010/63/EU) and the Spanish RD 53/2013. The experiments and procedures were performed approved by the Bioethical Committees of the University of Murcia (approval number #395/2017).

### CRISPRo, RNA, and recombinant protein injections in zebrafish

CRISPR RNA (crRNA) for zebrafish *ace2* (table S1) and negative control crRNA (catalog no. 1072544, crSTD), and tracrRNA (trans-activating crRNA; catalog no. 1072533) were purchased from Integrated DNA Technologies (IDT) and resuspended in nuclease-free duplex buffer to 100 μM. One microliter of each was mixed and incubated for 5 min at 95°C for duplexing. After removing from the heat and cooling to room temperature, 1.43 μl of nuclease-free duplex buffer was added to the duplex, giving a final concentration of 1000 ng/μl. Last, the injection mix was prepared by mixing 1 μl of duplex, 2.55 μl of nuclease-free duplex buffer, 0.25 μl of Cas9 nuclease V3 (IDT, catalog no. 1081058), and 0.25 μl of phenol red, giving final concentrations of gRNA duplex (250 ng/μl) and of Cas9 (500 ng/μl). The prepared mix was microinjected into the yolk of one- to eight-cell-stage embryos using a microinjector (Narishige) (0.5 to 1 nl per embryo). The same amounts of gRNA were used in all the experimental groups. The efficiency of gRNA was checked by amplifying the target sequence with a specific pair of primers (table S1) and the TIDE webtool (https://tide.nki.nl/).

In vitro–transcribed RNA was obtained following the manufacturer’s instructions (mMESSAGE mMACHINE kit, Ambion). RNA was mixed in microinjection buffer and microinjected into the yolk of one-cell-stage embryos using a microinjector (Narishige; 0.5 to 1 nl per embryo). The same amount of RNA was used for all the experimental groups.

Recombinant His-tagged S1 WT (#40591-V08B1), S1 (L18F, D80A, D215G, LAL242-244 deletion, R246I, K417N, E484K, N501Y, and D614G) (variant β, catalog no. 40591-V08H15), S1 [L18F, T20N, P26S, D138Y, R190S, K417T, E484K, N501Y, D614G, and H655Y (variant γ, catalog no. 40591-V08H14) and E154K, L452R, E484Q, D614G, and P681R (variant δ, catalog no. 40591-V08H19] (from Sino Biological), WT S1 (catalog no. RP01262), S1 + S2 (catalog no. RP01283LQ) and envelope protein (E; catalog no. RP01263) (from ABclonal), flagellin (Invivogen), or BSA (Sigma-Aldrich) at a concentration of 0.25 mg/ml supplemented with phenol red was injected into the hindbrain (1 nl). All S1 proteins were produced in baculovirus-insect cells (WT) or human embryonic kidney 293 cells (variants β, γ, and δ) and had <1.0 EU per μg protein as determined by the limulus amebocyte lysate method.

### Chemical treatments

Two–day postfertilization (dpf) larvae were manually dechorionated and treated for 1 or 4 hours at 28°C by bath immersion with the caspase-1 inhibitor VX-765 (Belnacasan, Selleckchem) at a final concentration of 100 μM diluted in egg water [ocean sea salt (60 μg/ml) dissolved in distilled water] supplemented with 0.1% dimethyl sulfoxide (DMSO). In some experiments, 24-hpf embryos were treated with 0.3% *N*-phenylthiourea (PTU; Sigma-Aldrich) to inhibit melanogenesis.

### Analysis of gene expression

Total RNA was extracted from whole larvae, head/tail larvae, or human cell pellets with TRIzol reagent (Invitrogen) following the manufacturer’s instructions and treated with deoxyribonuclease I, amplification grade (RNA, 1 U/mg; Invitrogen). SuperScript IV RNase H Reverse Transcriptase (Invitrogen) was used to synthesize first-strand cDNA with random primer from 1 μg of total RNA at 50°C for 50 min. Real-time PCR was performed with an ABIPRISM 7500 instrument (Applied Biosystems) using SYBR Green PCR Core Reagents (Applied Biosystems). Reaction mixtures were incubated for 10 min at 95°C, followed by 40 cycles of 15 s at 95°C, 1 min at 60°C, and, last, 15 s at 95°C, 1 min 60°C, and 15 s at 95°C. For each mRNA, gene expression was normalized to the *rps11* (zebrafish) or *ACTB* (human) content in each sample, using the Pfaffl method (*58*). The primers used are shown in table S1. In all cases, each PCR was performed with triplicate samples and repeated at least with two independent samples.

### Caspase-1 activity assays

The caspase-1 activity was determined with the fluorometric substrate Z-YVAD 7-amido-4-trifluoromethylcoumarin (Z-YVAD-AFC, caspase-1 substrate VI, Calbiochem), as described previously (*37*, *39*). Briefly, 25 to 35 larvae were lysed in hypotonic cell lysis buffer [25 mM 4-(2-hydroxyethyl) piperazine-1-ethanesulfonic acid, 5 mM ethylene glycol-bis(2-aminoethylether)-*N*,*N*,*N*′,*N*′-tetraacetic acid, 5 mM dithiothreitol, and 1:20 protease inhibitor cocktail (Sigma-Aldrich) (pH 7.5)] on ice for 10 min. For each reaction, 100 μg of protein was incubated for 90 min at room temperature with 50 mM YVAD-AFC and 50 μl of reaction buffer {0.2% 3-[(3-cholamidopropyl)dimethylammonio]-1-propanesulfonate, 0.2 M 4-(2-hydroxyethyl) piperazine-1-ethanesulfonicacid, 20% sucrose, and 29 mM dithiothreitol (pH 7.5)}. After incubation, the fluorescence of the AFC released from the Z-YVAD-AFC substrate was measured with a FLUOstart spectofluorometer (BGM, LabTechnologies) at an excitation wavelength of 405 nm and an emission wavelength of 492 nm. A representative caspase-1 activity graph out of three repeats is shown in figures.

### Imaging of zebrafish larvae

To study immune cell recruitment to the injection site and Nfkb activation, 2-dpf *mpx:eGFP*, *mfap4:mcherry*, or *nfkb:egfp* larvae were anesthetized in embryo medium with tricaine (0.16 mg/ml). Images of the hindbrain, head, or the whole-body area were taken 3, 6, 12, and 24 h hpi using a Leica MZ16F fluorescence stereomicroscope. The number of neutrophils or macrophages was determined by counting visually and the fluorescence intensity was obtained and analyzed with ImageJ (Fiji) software (*59*).

*fli1a:EGFP;gata1a:dsRED* larvae previously treated with PTU were mounted for imaging in 1.6% low-melting agarose in embryo medium with tricaine. Confocal images were acquired 24 hours after hindbrain injection with the different recombinant S1 proteins on a widefield microscope (Leica THUNDER imager) with a 10×/0.32 objective using a DFC 9000 GTC sCMOS camera (Leica, Weztlar). The images are a maximum projection of a *z*-stack of 27 planes spaced at 3.8 μm and were processed with the Leica large-volume computational clearing algorithm, which consists of a regularized Richardson-Lucy deconvolution preceded by a proprietary background subtraction (Leica ICC) that improves the deconvolution results on thick samples.

Dead cells in the injection site were visualized with acridine orange (AO; Sigma-Aldrich). Anesthetized larvae were incubated with AO (10 μg/ml) in egg water for 30 min, washed three times for 10 min in egg water, and immediately visualized in a stereomicroscope using a green fluorescence filter. In all experiments, images were pooled from at least three independent experiments performed by two people and using blinded samples.

### Human PBMCs, neutrophils, and synovial fluid cell culture and treatments

All the experiments and procedures were performed as approved by the Ethical Clinical Research Committee of The University Hospital Virgen de la Arrixaca (approval number #5/2021). Peripheral blood was obtained from healthy donors and diluted one-half with physiological serum. The diluted blood was added to Lymphoprep (Alere Technologies AS, catalog no. 1114545) avoiding phase mixing. Subsequently, the tube was centrifuged for 30 min at 400*g* without brake. PBMCs were collected from the white phase and neutrophils from the pellet of the sample. PBMCs were then washed once with RPMI medium supplemented with 1% penicillin/streptomycin, 1% glutamine, and 10% fetal bovine serum and centrifuged for 5 min at 400*g*. Last, cells were resuspended in supplemented RPMI medium to reach a final concentration of 10^6^ cells/ml. Neutrophils were washed with ACK lysis buffer (catalog no. 0000944064) to eliminate the erythrocytes and then twice with phosphate-buffered saline. The remaining cells were resuspended in supplemented RPMI medium to a final concentration of 10^6^ cells/ml. The synovial fluid, obtained from a 13-year-old female with recent onset of oligoarticular juvenile idiopathic arthritis, was composed of 87% mononuclear cells and 13% neutrophils. The fluid was treated with hyaluronidase for 10 min at room temperature and then centrifuged for 10 min at 400*g*. The cell pellet was washed with ACK lysis buffer and then resuspended in supplemented RPMI medium to a final concentration of 10^6^ cells/ml. Cells were seeded at approximately 4 × 10^5^ cells per well in a 24-well plate and stimulated with recombinant S1, S1 + S2, and E proteins (1 μg/ml) for 4 hours at 37°C. As controls, flagellin and the E protein preheated at 80°C for 30 min were used at the same concentration.

### Statistical analysis

The statistical differences among groups were determined by an analysis of variance (ANOVA) and a Tukey multiple range test were. The differences between two samples were analyzed by Student’s *t* test. In some experiments, a Fisher’s exact test was used.

## Notes

View/request a protocol for this paper from *Bio-protocol*.
