# Supplementary material for: Differential proinflammatory activities of Spike proteins of SARS-CoV-2 variants of concern
Source: Sci Adv. 2022 Sep 16;8(37):eabo0732. doi: 10.1126/sciadv.abo0732 (PMC9481140; doi:10.1126/sciadv.abo0732)
Supplement: Supplementary file 1 — Figs. S1 to S11 Table S1 [file sciadv.abo0732_sm.pdf]

Science Advances
AAAS

Supplementary Materials for

Differential proinflammatory activities of Spike proteins of SARS-CoV-2 variants of concern

Sylwia D. Tyrkalska *et al.*

Corresponding author: Sylwia D. Tyrkalska, tyrkalska.sylwia@gmail.com;
María L. Cayuela, marial.cayuela@carm.es; Victoriano Mulero, vmulero@um.es

Sci. Adv. **8**, eabo0732 (2022)
DOI: 10.1126/sciadv.abo0732

This PDF file includes:

Figs. S1 to S11
Table S1

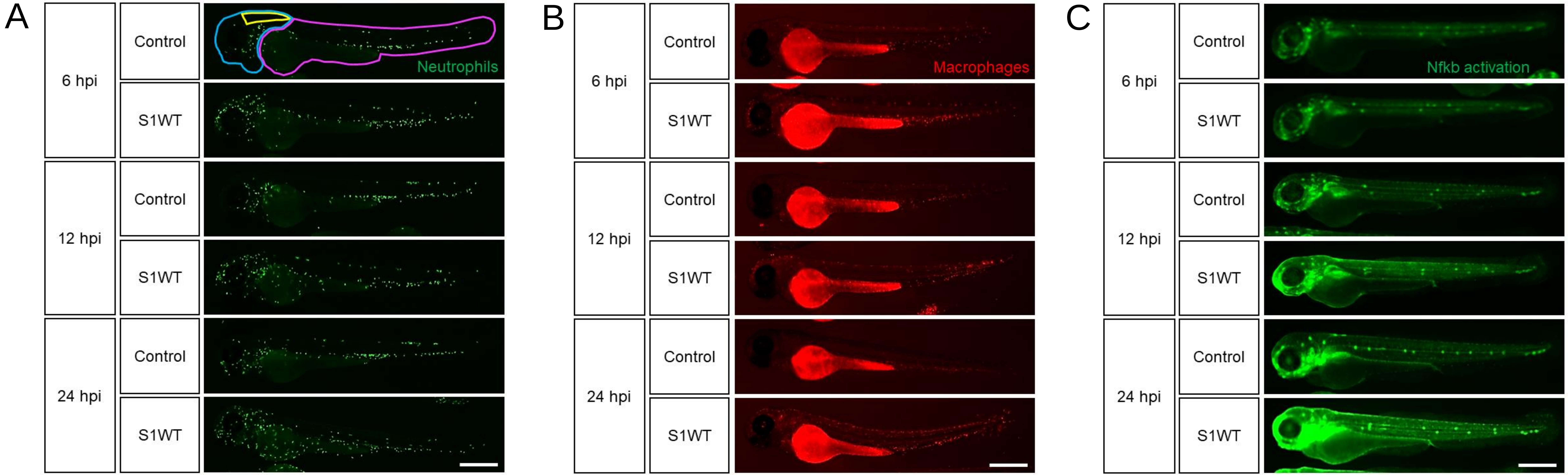

Figure S1 (related to Figure 1). Representative green and red fluorescence images of larvae of the different groups shown in Figures 1A-1C. Recombinant S1WT or vehicle (-) were injected in the hindbrain ventricle (HBV) of 2 dpf *Tg(mpx:eGFP)* (A), *Tg(mfap4:mCherry)* (B) and *Tg(NFkB-RE:eGFP)* (C) and neutrophils (A), macrophages (B) and Nfkb activation (C) visualized at 6, 12 and 24 hpi by fluorescence microscopy. The regions of interest used for quantitation in all experiments are indicated in A: hindbrain (yellow), head (blue) and rest of the body (violet). Bars: 500 µm.

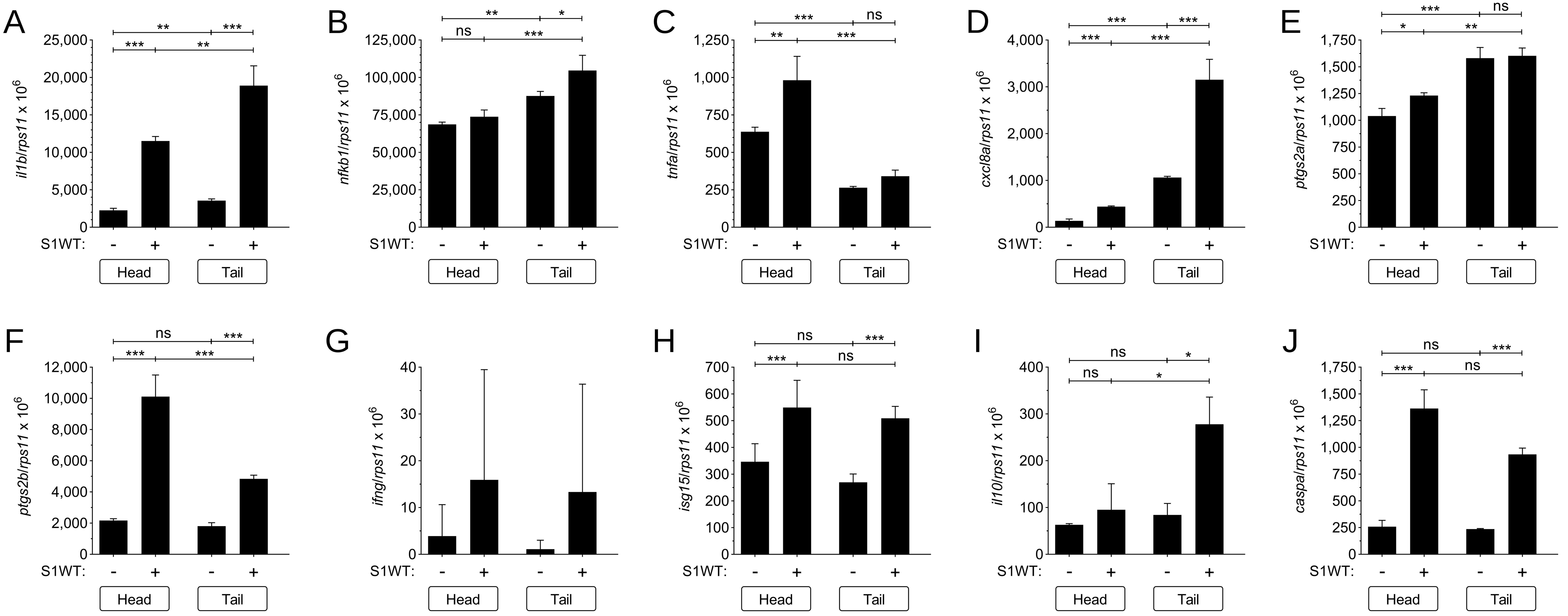

Figure S2 (related to Figure 1): Gene expression analysis of zebrafish larvae injected with wild type S1. Recombinant S1WT or vehicle (-) were injected in the hindbrain ventricle (HBV) of 2 dpf wild type larvae and the transcript levels of the indicated genes were analyzed at 12 hpi by RT-qPCR in larval head and tail. Data are shown as mean + S.E.M. P values were calculated using one-way ANOVA and Tukey multiple range test. ns, not significant, *≤p0.05, **p≤0.01, ***p≤0.001.

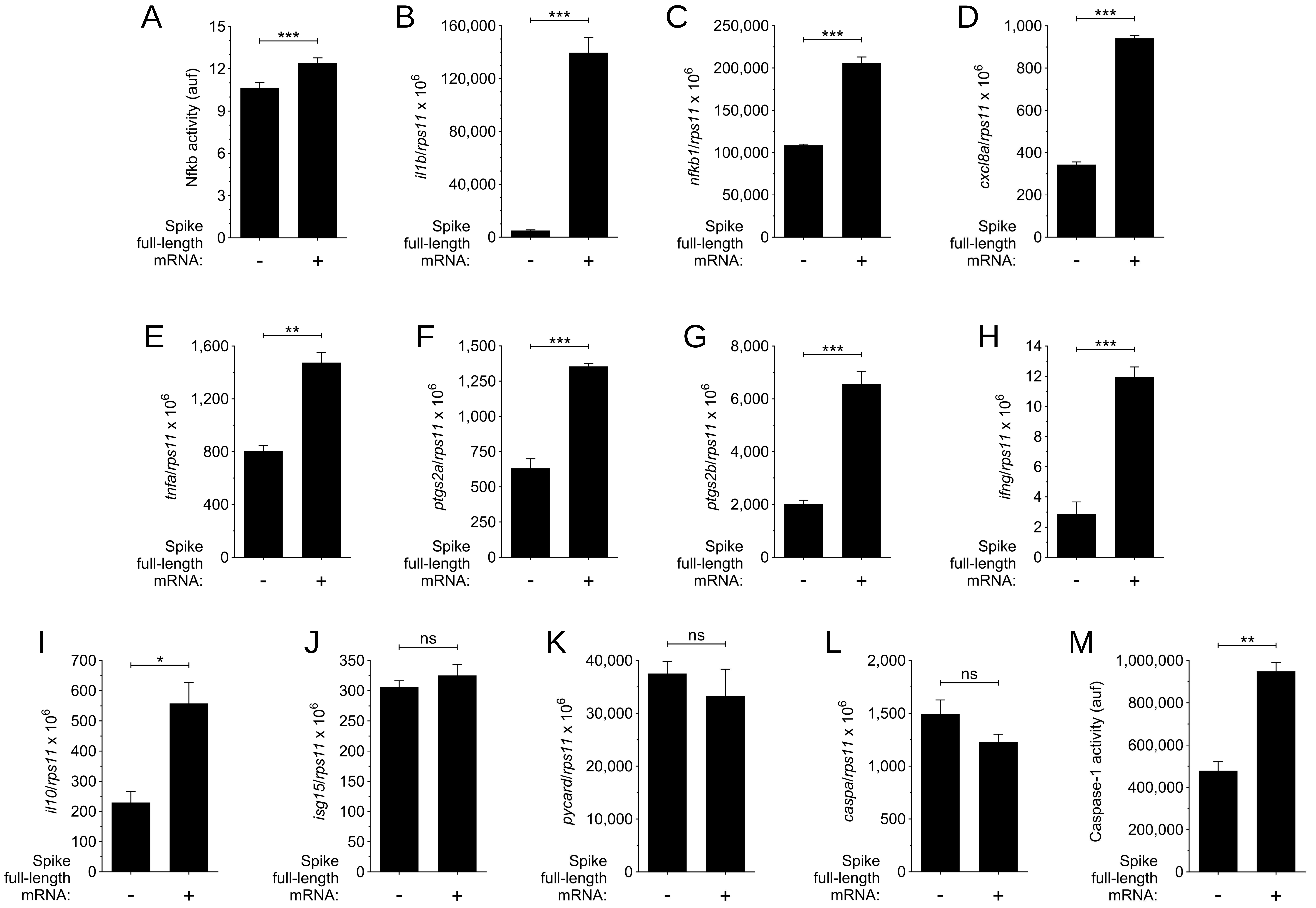

Figure S3 (related to Figure 1). Full-length S protein phenocopies the effects of recombinant S1 protein in zebrafish. One-cell stage zebrafish eggs of *Tg(NFkB-RE:eGFP)* (A) or wild type (B-M) were microinjected with control (antisense) or full-length S RNAs. Nfkb activation was analyzed at 48 hpf by fluorescence microscopy (A), the transcript levels of the indicated genes were analyzed at 48 hpf by RT-qPCR (B-L), and caspase-1 activity was determined at 72 hpf using a fluorogenic substrate (M). Data are shown as the mean ± S.E.M. obtained from 3 replicates. P values were calculated using one-way ANOVA followed by Tukey multiple range test. ns, not significant, *≤p0.05, **p≤0.01, ***p≤0.001.

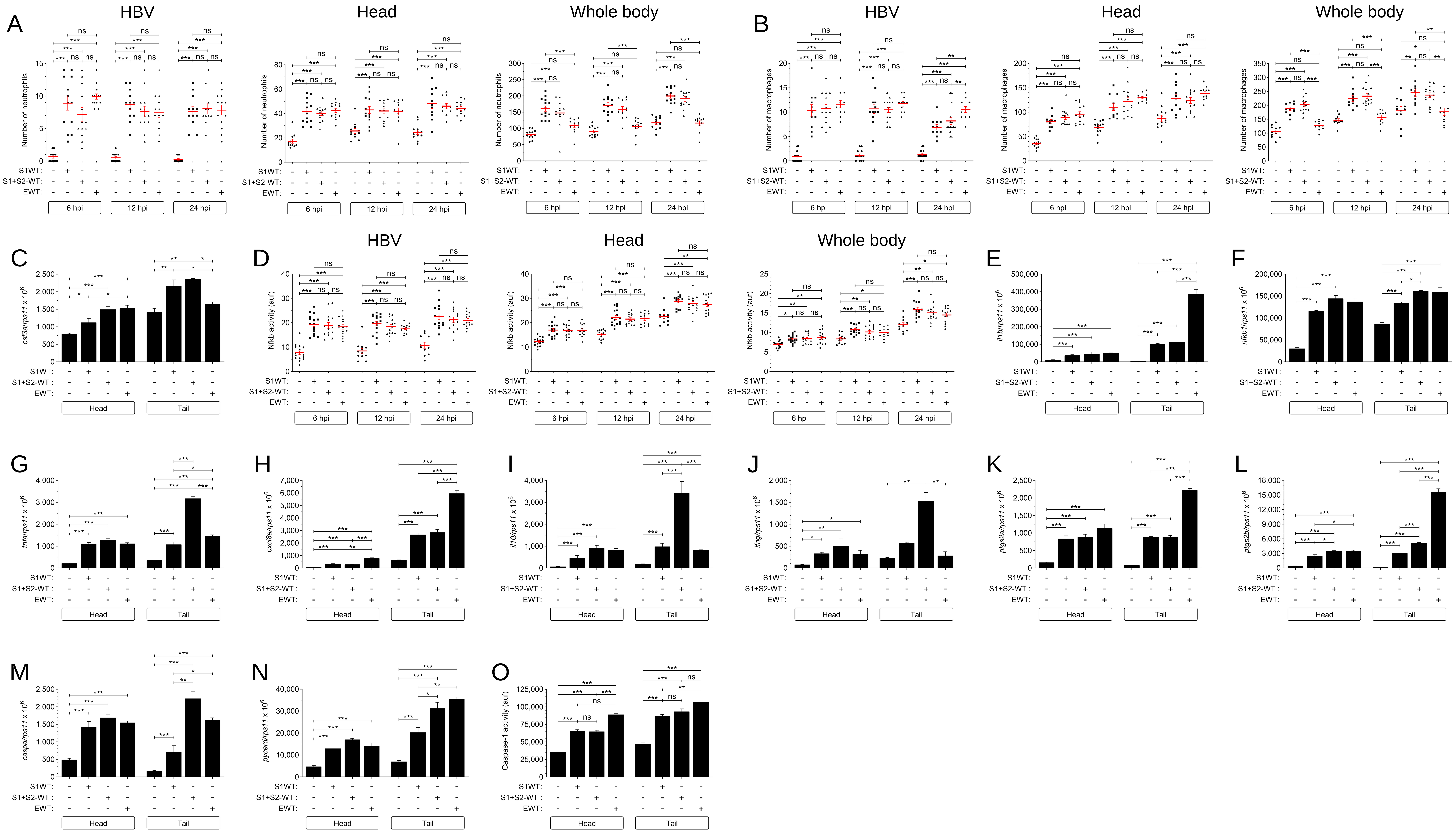

Figure S4 (related to Figure 1). Recombinant S1, S1+S2 and E proteins are all proinflammatory in zebrafish. Recombinant S1, S1+S2, E or vehicle (-) from wild type SARS-CoV-2 were injected in the hindbrain ventricle (HBV) of 2 dpf larvae of *Tg(mpx:eGFP)* (A), *Tg(mfap4:mCherry)* (B), *Tg(NFkB-RE:eGFP)* (D) and wild type (C, E-O). Neutrophil (A) and macrophage (B) recruitment and number, and Nfkb activation (D) were analyzed at 6, 12 and 24 hpi by fluorescence microscopy, the transcript levels of the indicated genes were analyzed at 12 hpi by RT-qPCR (C, E-N), and caspase-1 activity was determined at 24 hpi using a fluorogenic substrate (O). Each dot represents one individual and the mean ± S.E.M. for each group is also shown. P values were calculated using one-way ANOVA and Tukey multiple range test. ns, not significant, *≤p0.05, **p≤0.01, ***p≤0.001.

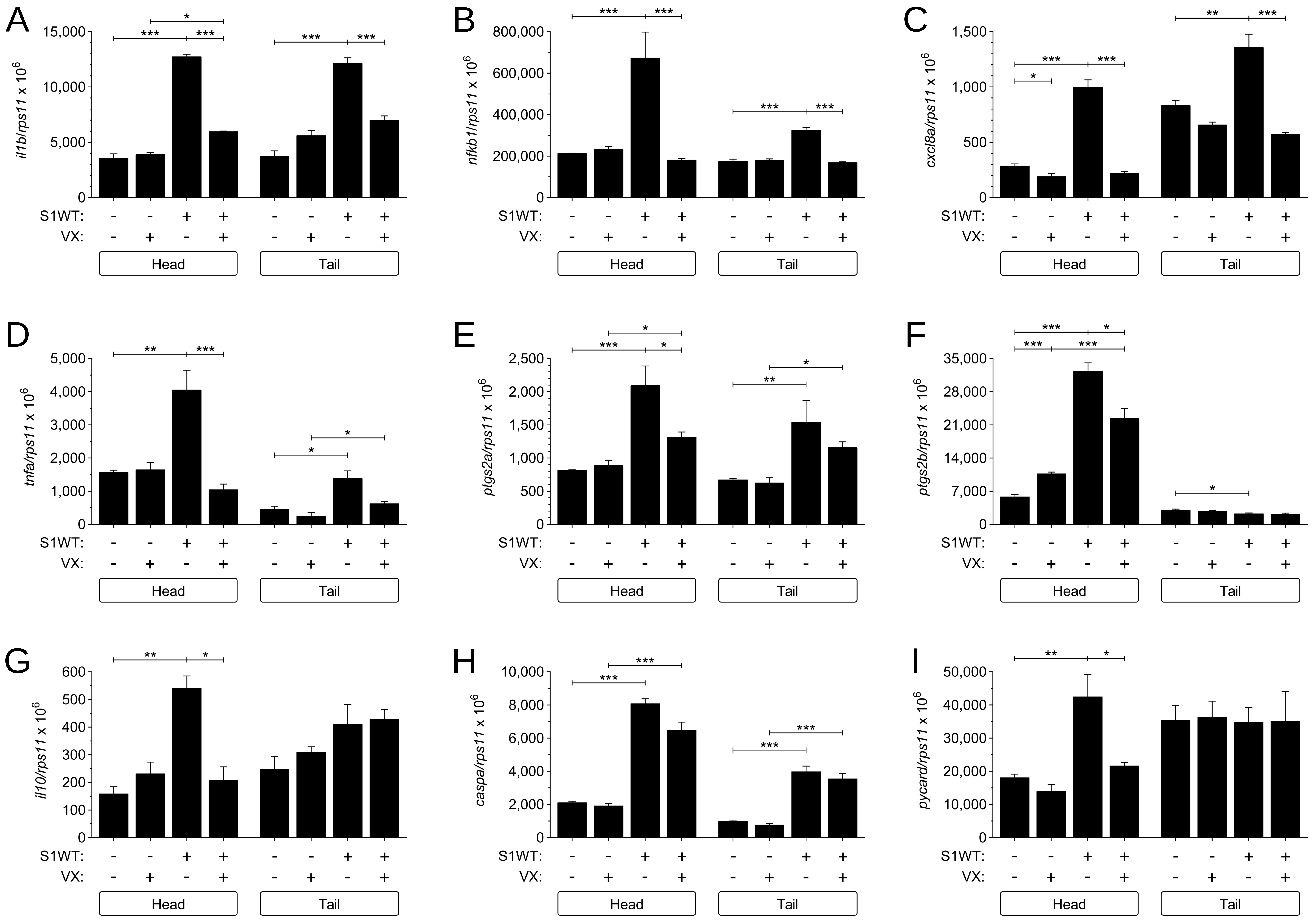

Figure S5 (related to Figure 2): Gene expression analysis of zebrafish larvae injected with wild type S1 upon pharmacological inhibition of the inflammasome. Recombinant S1WT or vehicle (-) were injected in the hindbrain ventricle (HBV) of 2 dpf wild type larvae in the presence of either DMSO or the caspase-1 inhibitor VX-765 (VX). The transcript levels of the indicated genes were analyzed at 12 hpi by RT-qPCR in larval head and tail. Data are shown as mean + S.E.M. P values were calculated using one-way ANOVA and Tukey multiple range test. *≤p0.05, **p≤0.01, ***p≤0.001.

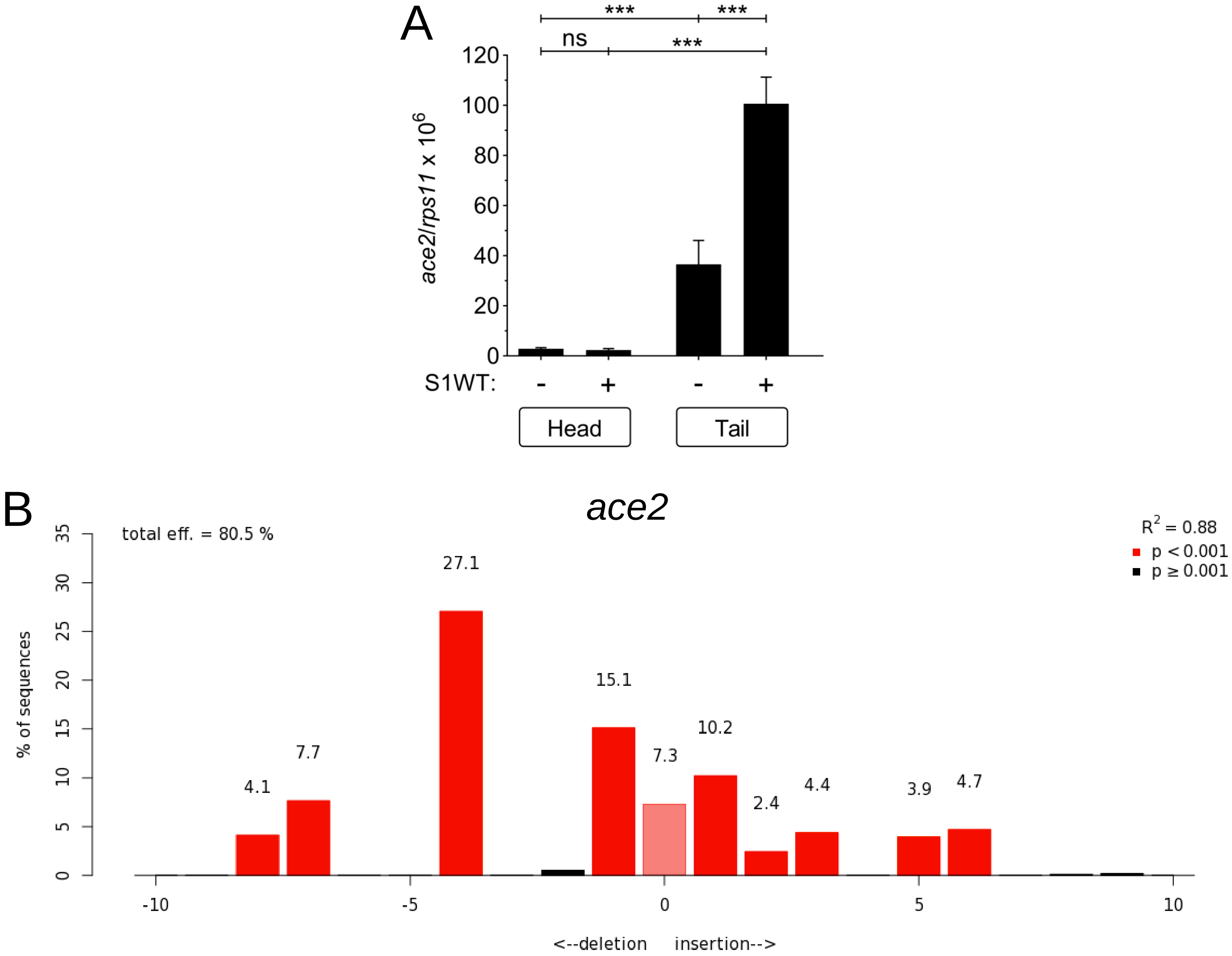

Figure S6 (related to Figure 3). Expression and efficiency of the crRNA of *ace2*. (A) Recombinant S1WT or vehicle (-) were injected in the hindbrain ventricle (HBV) of 2 dpf wild type larvae. The transcript levels of *ace2* were analyzed at 12 hpi by RT-qPCR in larval head and tail. P values were calculated using one-way ANOVA and Tukey multiple range test. ns, not significant, *≤p0.05, **p≤0.01. (B) Analysis of genome editing efficiency (80.5%) in larvae injected with ace2 crRNA/Cas9 complexes and quantification rate of nonhomologous end joining-mediated repair showing all insertions and deletions (INDELS) at the target site (https://tide.nki.nl/).

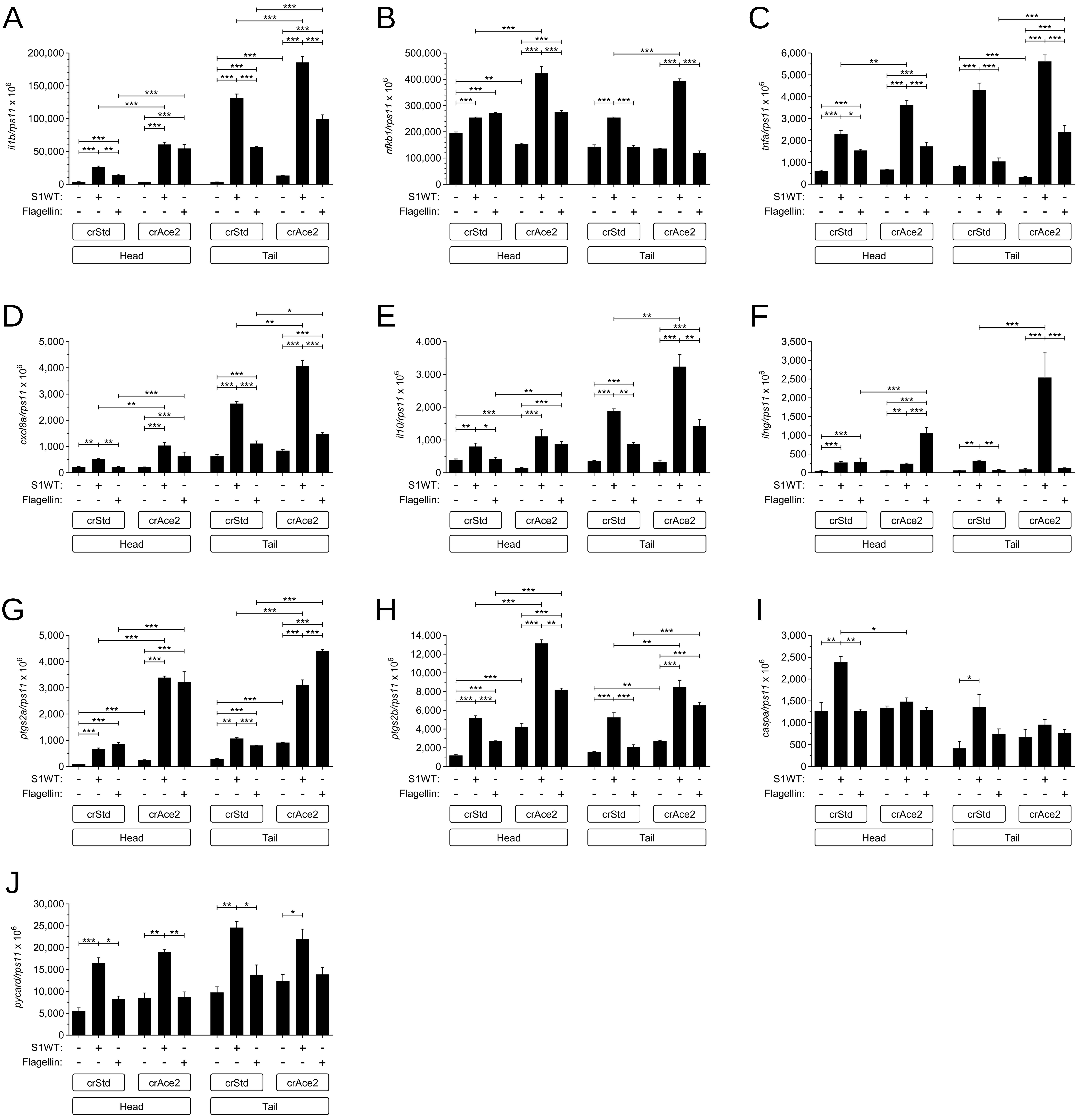

Figure S7 (related to Figure 3): Gene expression analysis of zebrafish larvae injected with wild type S1 upon manipulation of the Ace2/Ang (1-7) axis. One-cell stage embryos were microinjected with control or *ace2* crRNA/Cas9 complexes. At 2 dpf, recombinant S1WT, flagellin or vehicle (-) were injected alone or in combination with Ang (1-7) in the hindbrain ventricle (HBV) of control and Ace2-deficient larvae. The transcript levels of the indicated genes were analyzed at 12 hpi by RT-qPCR in larval head and tail. Data are shown as mean + S.E.M. P values were calculated using one-way ANOVA and Tukey multiple range test. *≤p0.05, **p≤0.01, ***p≤0.001.

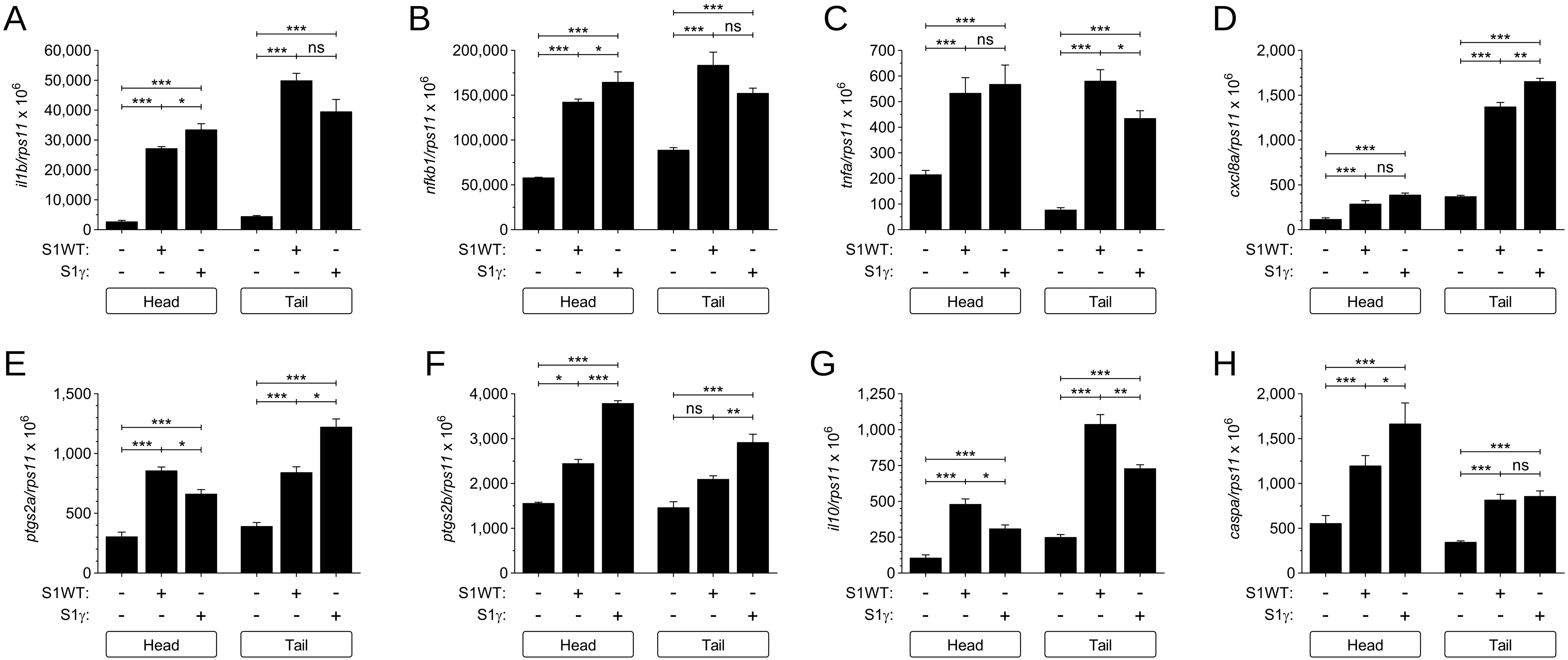

Figure S8 (related to Figure 4): Gene expression analysis of zebrafish larvae injected with S1γ. Recombinant S1γ or vehicle (-) were injected in the hindbrain ventricle (HBV) of 2 dpf wild type larvae and the transcript levels of the indicated genes were analyzed at 12 hpi by RT-qPCR in larval head and tail. Data are shown as mean + S.E.M. P values were calculated using one-way ANOVA and Tukey multiple range test. ns, not significant, *≤p0.05, **p≤0.01, ***p≤0.001.

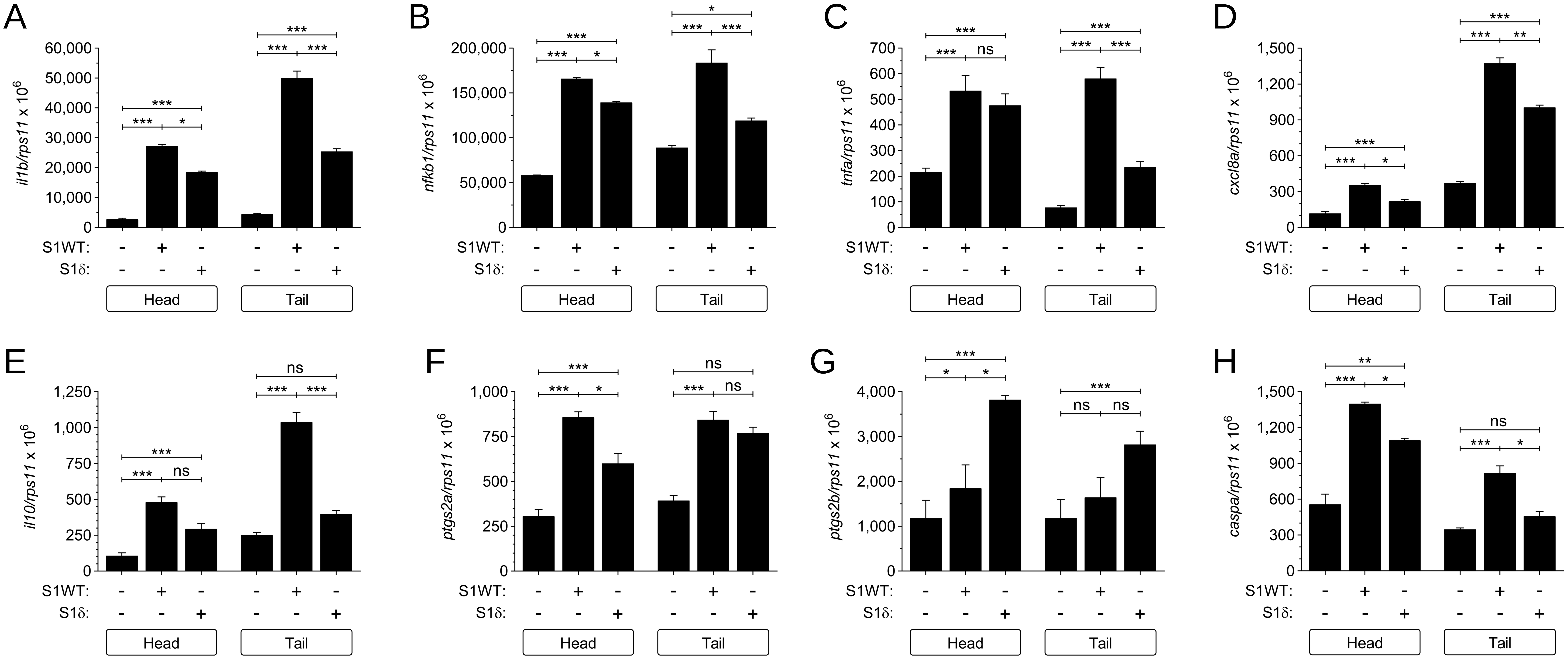

Figure S9 (related to Figure 5): Gene expression analysis of zebrafish larvae injected with S1δ. Recombinant S1δ or vehicle (-) were injected in the hindbrain ventricle (HBV) of 2 dpf wild type larvae and the transcript levels of the indicated genes were analyzed at 12 hpi by RT-qPCR in larval head and tail. Data are shown as mean + S.E.M. P values were calculated using one-way ANOVA and Tukey multiple range test. ns, not significant, *≤p0.05, **p≤0.01, ***p≤0.001.

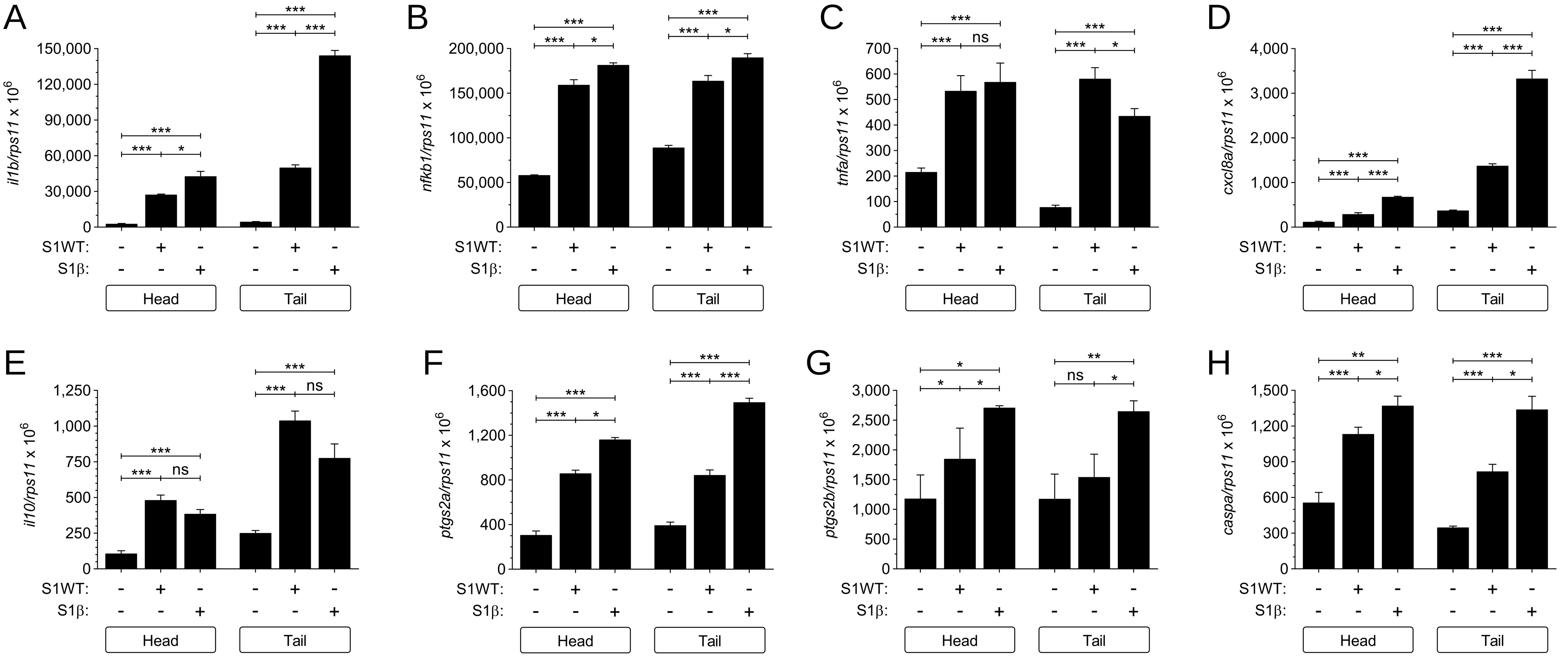

Figure S10 (related to Figure 6): Gene expression analysis of zebrafish larvae injected with S1β. Recombinant S1β or vehicle (-) were injected in the hindbrain ventricle (HBV) of 2 dpf wild type larvae and the transcript levels of the indicated genes were analyzed at 12 hpi by RT-qPCR in larval head and tail. Data are shown as mean + S.E.M. P values were calculated using one-way ANOVA and Tukey multiple range test. ns, not significant, *≤p0.05, **p≤0.01, ***p≤0.001.

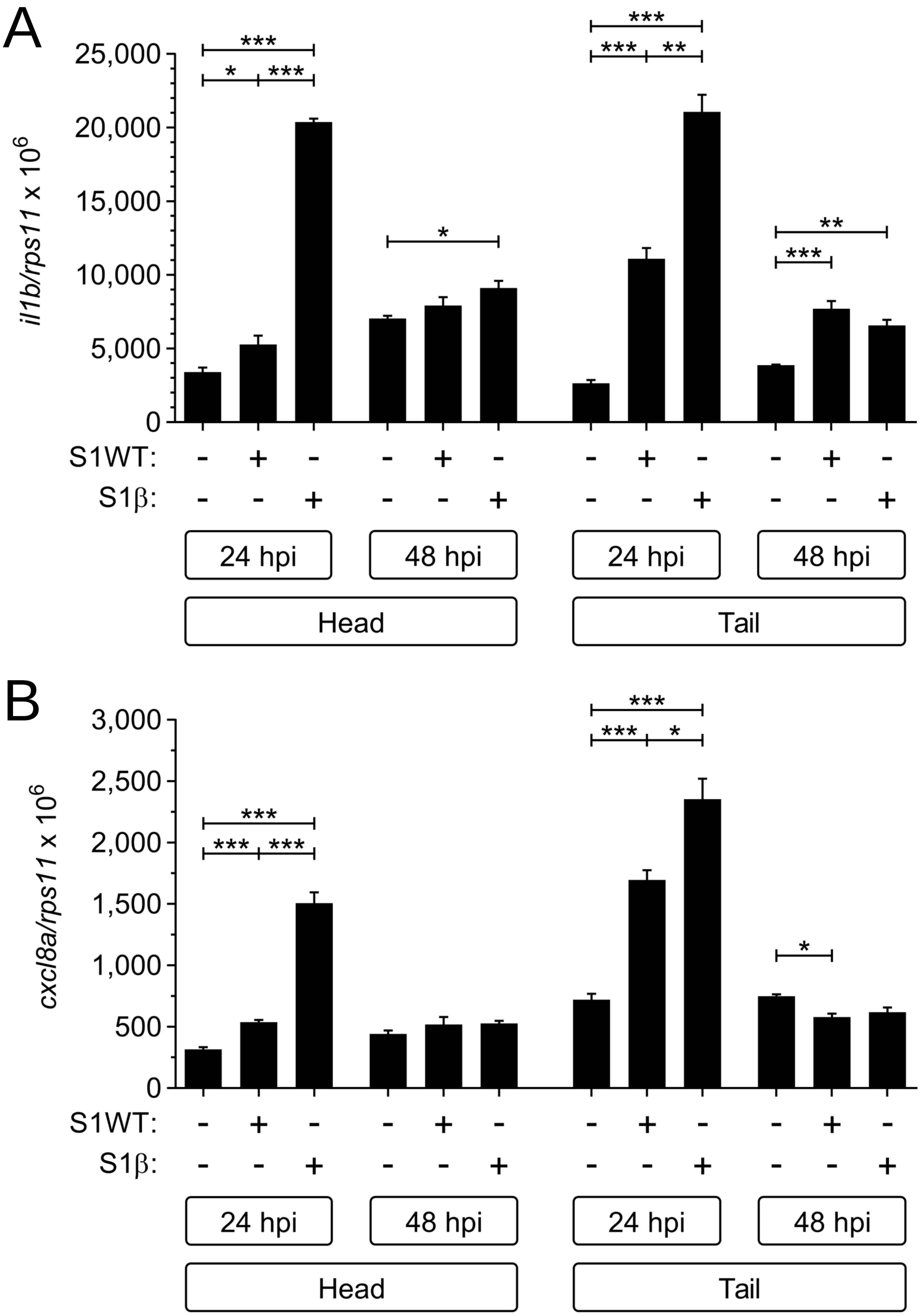

Figure S11 (related to Figure 6). S1β shows delayed but stronger proinflammatory activity than S1WT. Recombinant S1WT, S1β or vehicle (-) were injected in the hindbrain ventricle (HBV) of 2 dpf wild type larvae. The transcript levels of *il1b* (A) and *cxcl8a* (B) were analyzed at 24 and 48 hpi by RT-qPCR. The data are shown as the mean ± S.E.M from 3 replicates. P values were calculated using one-way ANOVA and Tukey multiple range test. ns, not significant, *≤p0.05, **p≤0.01, ***p≤0.001.

Table S1. Primers and crRNAs used in this study. The gene symbols followed the Zebrafish Nomenclature Guidelines (https://zfin.atlassian.net/wiki/spaces/general/pages/1818394635/ZFIN+Zebrafish+Nomenclature+Conventions). Ena, European Nucleotide Archive.

Gene	ENA or ENSEMBL accesion number	Name	Sequence (5'→3')	Use
rps11	NM_213377.1	F	ACAGAAATGCCCCTTCACTG	RT-qPCR
		R	GCCTCTTCTCAAAACGGTTG	
il1b	NM_212844.2	F	GCCTGTGTGTTTGGGAATCT	
		R	TGATAAACCAACCGGGACA	
nfkb1	ENSDARG00000105261.2	F	TTCTTCTTGGTCACGTGCAG	
		R	ACTCTCAGCATCCGCATCTT	
tnfa	NM_212859.2	F	GCGCTTTTCTGAATCCTACG	
		R	TGCCCAGTCTGTCTCCTTCT	
cxcl8a	XM_001342570.7	F	GTCGCTGCATTGAAACAGAA	
		R	CTTAACCCATGGAGCAGAGG	
il10	NM_001020785.2	F	AACTCAAGCGGGATATGGTG	
		R	ATCAAGCTCCCCCATAGCTT	
ifng1r (*ifng*)	NM_001020793.1	F	CTTCAGACAACCAGCGCATA	
		R	TTTTCCAACCCAATCCTTTG	
ptgs2a	NM_153657.1	F	TGGATCTTTCCTGGTGAAGG	
		R	GAAGCTCAGGGGTAGTGCAG	
ptgs2b	NM_001025504.2	F	CCCTCATGCCTGATGATTTT	
		R	CCACCCTTAACACTGCTGGT	
caspa	NM_131505.2	F	CGACGTCAGGGAGATAAGGC	
		R	TGGATACTAAGGTTTTGAACGACG	
pycard	NM_131495.2	F	ATTTTGAGGGCGATCAAGTG	
		R	GCATCCTCAAGGTCATCCAT	
csf3a	NM_001145242.1	F	CCGCGGCCTCAGTCTGGAAA	
		R	TGAAGCAACGACCCTGTCGCA	
isg15	ENSDART00000130554.3	F	TTACCATCATAACTCGGTGA	
		R	ACAGCTTCTGCTTAAAAGTG	
ace2	NM_001007297.1	F	ACAAAGCTAGTGAACTGAGA	
		R	CCGTGTTGTAAATAGTGCTC	
ace2	NM_001007297.1	F	AGAGGCATATGCAATCTGGAGC	Target sequence amplification from gDNA
		R	ATACATCTGGCTCTTTAAACTCACA	
ace2	NM_001007297.1	Dr.Cas9.ACE2.1.AC	CCGATTGTCAGACTTTGGAG	CRISPR-Cas9
